# Supplementary material for: A fast and scalable framework for large-scale and ultrahigh-dimensional sparse regression with application to the UK Biobank
Source: PLoS Genet. 2020 Oct 23;16(10):e1009141. doi: 10.1371/journal.pgen.1009141 (PMC7641476; doi:10.1371/journal.pgen.1009141)
Supplement: S1 Appendix — (PDF) [file pgen.1009141.s001.pdf]

S1 Appendix. Results for Body Mass Index (BMI) BMI is another polygenic trait that is widely studied. Like height, it is heritable and easily measured. It is also a trait of interest, since obesity is a risk factor for diseases such as type 2 diabetes and cardiovasclar disease. Recent studies estimate heritability at 0.42 [33, 34] and 27% of the variance can be explained using a genomic model [36]. We expect the heritability to be lower than that for height, since intuitively speaking, one component of the body mass, weight, should heavily depend on environmental factors, for example, individual's lifestyle. From GWAS studies, 97 associated loci have been identified, but they only account for 2.7% of the variance [57, 58]. Although the estimates of heritability are not precise, there may be more missing heritability for BMI than for height. We also find lower R^2 values using the lasso. The results for models fitted on the training set and selected based on the validation performance (without refitting) are summarized in S1 Table. The R^2 curves for the lasso and the relaxed lasso are shown in S1 Figure. From the table, we see that more than 26,000 variants are selected by the lasso to attain an R^2 greater than 10%. In constrast, the relaxed lasso and the sequential linear regression use around one-tenths of the variables, and end up with degraded predictive performance both at around 5%. From S2 Figure, we see further evidence that the actual BMI is of high variability and hard to predict with the lasso model — the correlation between the predicted value and the actual value is 0.3256. From S3 Figure, we also see the distribution is skewed to the right, suggesting a number of exceedingly high observed values than the ones predicted by the model. Nevertheless, we are able to predict BMI within 9 kg/m^2 about 95% of the time.

With the refitting mechanism, from S2 Table, we see that the elastic-net and ridge regression does the best job compared with other well-established PRS methods, followed by SBayesR.
