# Supplementary material for: A fast and scalable framework for large-scale and ultrahigh-dimensional sparse regression with application to the UK Biobank
Source: PLoS Genet. 2020 Oct 23;16(10):e1009141. doi: 10.1371/journal.pgen.1009141 (PMC7641476; doi:10.1371/journal.pgen.1009141)
Supplement: S2 Appendix — (PDF) [file pgen.1009141.s002.pdf]

S2 Appendix. Results for Asthma Asthma is a common respiratory disease characterized by inflammation of airways in the lungs and difficulty breathing. It is another complex, polygenic trait that is associated with both genetic and environmental factors. Our results for models fitted on the training set and selected based on the validation performance (without refitting) are summarized in S3 Table. The AUC curves for the lasso and the relaxed lasso are shown in S4 Figure. In addition, for each test sample, we compute the percentile of its predicted score/probability among the entire test cohort, and create box plots of such percentiles separately for the control group and the case group. We see in S5 Figure that there is a significant overlap between the box plots of the two groups, suggesting that asthma is difficult to predict. This can also be seen from the AUC value and the ROC curve in S10 Figure. That being said, the multivariate lasso still does much better than the baseline model and the strongest univariate model. In S6 Figure, we stratify the prediction percentile into 10 bins, and compute the overall prevalence within each bin. We observe a clear upward trend that provides further evidence that we manage to capture some genetic signal there.

Under refitting, we see from S4 Table that SBayesR achieves the best AUC for asthma among these PRS methods, though the difference is not significant compared with the refit lasso, elastic-net and ridge regression.
