# Supplementary material for: A fast and scalable framework for large-scale and ultrahigh-dimensional sparse regression with application to the UK Biobank
Source: PLoS Genet. 2020 Oct 23;16(10):e1009141. doi: 10.1371/journal.pgen.1009141 (PMC7641476; doi:10.1371/journal.pgen.1009141)
Supplement: S3 Appendix — (PDF) [file pgen.1009141.s003.pdf]

S3 Appendix. Results for High Cholesterol. High cholesterol is characterized by high amounts of cholesterol present in the blood and is a risk factor for cardiovascular disease. It is highly heritable and may be polygenic. Our results for models fit on the training set and selected based on the validation performance (without refitting) are summarized in S5 Table . The AUC curves for the lasso and the relaxed lasso are shown in S7 Figure. Similarly the ROC curve for the best lasso model is shown in S11 Figure, and box plots for the two groups and a stratified prevalence plot are shown in S8 Figure and S9 Figure. We see that the distributions of predictions made on non-HC individuals and on HC individuals are clearly different from each other, suggesting good classification results. That is reflected in the AUC measure listed in the table. Nevertheless, it is not much better than the result of the base model including only covariates age and sex.

Under refitting, we see from S6 Table that SBayesR achieves the best AUC for high cholesterol among these PRS methods, though the difference is not significant compared with the refit lasso/elastic-net.
