# Supplementary material for: A fast and scalable framework for large-scale and ultrahigh-dimensional sparse regression with application to the UK Biobank
Source: PLoS Genet. 2020 Oct 23;16(10):e1009141. doi: 10.1371/journal.pgen.1009141 (PMC7641476; doi:10.1371/journal.pgen.1009141)
Supplement: S4 Appendix — (PDF) [file pgen.1009141.s004.pdf]

S4 Appendix. Manhattan Plots.

The Manhattan plots in S12 Figure and S13 Figure (generated using the **qqman** package [59]) show the magnitude of the univariate p-values and the size of the lasso coefficients for each gene for the two quantitative traits and two binary traits. The coefficients in S14 Figure and S15 Figure are plotted for the model with the optimal R^2 value on the validation set. The variants highlighted in green in both plots are those that have coefficient magnitudes above the 99th percentile of all coefficient magnitudes for the trait. The horizontal line in the p-value plot is plotted at the genome-wide Bonferroni corrected p-value threshold 5×10^{-8}. There are two main points we would like to highlight:

- The lasso manages to capture significant univariate predictors in each genetic region. Due to possible correlation it does not pick up the variants with similarly small p-values located nearby.
- Some of the variants with weak univariate signals are also identified and turn out to be crucial to the predictive performance of the lasso.

For the two qualitative traits plotted in S16 Figure, S17 Figure, S18 Figure and S19 Figure, there are fewer p-values above the threshold, and many of the significant ones are located close to each other. The size of the lasso fit is correspondingly smaller, and the large coefficients pick up the important locations as before. However, the nonzero coefficients are still spread across the whole genome.
