# Supplementary material for: A fast and scalable framework for large-scale and ultrahigh-dimensional sparse regression with application to the UK Biobank
Source: PLoS Genet. 2020 Oct 23;16(10):e1009141. doi: 10.1371/journal.pgen.1009141 (PMC7641476; doi:10.1371/journal.pgen.1009141)
Supplement: S1 Table — R2 values for BMI (without refitting). For lasso and relaxed lasso, the chosen model is based on maximum R2 on the validation set. Model (3) to (9) each includes Model (2) plus their own specification as stated in the Form column. The elastic-net picks α = 0.1 based on the validation performance. (PDF) [file pgen.1009141.s005.pdf]

Model	Form	R^2_{train}	R^2_{val}	R^2_{test}	Size
(1)	Age + Sex	0.0092	0.0089	0.0083	2
(2)	Age + Sex + 10 PCs	0.0104	0.0103	0.0099	12
(3)	(2) + Single SNP	0.0134	0.0128	0.0124	13
(4)	(2) + 10K Combined	0.0384	0.0195	0.0210	10,012
(5)	(2) + 100K Combined	0.1307	0.0064	0.0093	100,012
(6)	Sequential LR	0.0865	0.0385	0.0395	2,012
(7)	Lasso	0.3196	0.1017	0.1052	26,060
(8)	Relaxed Lasso	0.1609	0.0504	0.0537	2,585
(9)	Elastic Net	0.4714	0.1121	**0.1151**	83,500
