# Supplementary material for: A fast and scalable framework for large-scale and ultrahigh-dimensional sparse regression with application to the UK Biobank
Source: PLoS Genet. 2020 Oct 23;16(10):e1009141. doi: 10.1371/journal.pgen.1009141 (PMC7641476; doi:10.1371/journal.pgen.1009141)
Supplement: S2 Table — (PDF) [file pgen.1009141.s006.pdf]

Model	Form	R^2_{test}	Size
(1)	Lasso	0.1186	23,338
(2)	Elastic Net	0.1287	97,012
(3)	Ridge	**0.1299**	118,012
(4)	PRS-CS	0.0315	148,064
(5)	SBayesR	0.1251	667,057
(6)	P + T	0.0455	6,547
(7)	Clumping	0.0535	7,745
