# Supplementary material for: A fast and scalable framework for large-scale and ultrahigh-dimensional sparse regression with application to the UK Biobank
Source: PLoS Genet. 2020 Oct 23;16(10):e1009141. doi: 10.1371/journal.pgen.1009141 (PMC7641476; doi:10.1371/journal.pgen.1009141)
Supplement: S3 Table — For lasso and relaxed lasso, the chosen model is based on maximum AUC on the validation set. Model (3) to (9) each includes Model (2) plus their own specification as stated in the Form column. The elastic-net picks α = 0.1 based on the validation performance. (PDF) [file pgen.1009141.s007.pdf]

Model	Form	AUC_{train}	AUC_{val}	AUC_{test}	Size
(1)	Age + Sex	0.5293	0.5297	0.5320	2
(2)	Age + Sex + 10 PCs	0.5342	0.5344	0.5367	12
(3)	(2) + Single SNP	0.5463	0.5476	0.5454	13
(4)	(2) + 10K Combined	0.5783	0.5580	0.5531	10,012
(5)	(2) + 100K Combined	0.6884	0.5644	0.5580	100,012
(6)	Sequential LR	0.6601	0.5883	0.5884	2,012
(7)	Lasso	0.7692	0.6159	0.6126	5,936
(8)	Relaxed Lasso	0.6747	0.5988	0.5955	621
(9)	Elastic Net	0.7803	0.6167	**0.6131**	7,811
