# Supplementary material for: A fast and scalable framework for large-scale and ultrahigh-dimensional sparse regression with application to the UK Biobank
Source: PLoS Genet. 2020 Oct 23;16(10):e1009141. doi: 10.1371/journal.pgen.1009141 (PMC7641476; doi:10.1371/journal.pgen.1009141)
Supplement: S4 Table — (PDF) [file pgen.1009141.s008.pdf]

Model	Form	AUC_{test}	Size
(1)	Lasso	0.6213	3,817
(2)	Elastic Net	0.6213	3,876
(3)	Ridge	0.6165	34,012
(4)	PRS-CS	0.5837	148,064
(5)	SBayesR	**0.6320**	667,057
(6)	P + T	0.5641	2,221
(7)	Clumping	0.5743	2,422
