# Supplementary material for: A fast and scalable framework for large-scale and ultrahigh-dimensional sparse regression with application to the UK Biobank
Source: PLoS Genet. 2020 Oct 23;16(10):e1009141. doi: 10.1371/journal.pgen.1009141 (PMC7641476; doi:10.1371/journal.pgen.1009141)
Supplement: S5 Table — For lasso and relaxed lasso, the chosen model is based on maximum AUC on the validation set. Model (3) to (9) each includes Model (2) plus their own specification as stated in the Form column. The elastic-net picks α = 0.9 based on the validation performance. (PDF) [file pgen.1009141.s009.pdf]

Model	Form	AUC_{train}	AUC_{val}	AUC_{test}	Size
(1)	Age + Sex	0.6918	0.6952	0.6883	2
(2)	Age + Sex + 10 PCs	0.6927	0.6959	0.6889	12
(3)	(2) + Single SNP	0.6963	6982	0.6921	13
(4)	(2) + 10K Combined	0.7402	0.6956	0.6880	10,012
(5)	(2) + 100K Combined	0.8518	0.6607	0.6547	100,012
(6)	Sequential LR	0.7540	0.7167	0.7137	1,012
(7)	Lasso	0.7832	0.7259	**0.7191**	4,277
(8)	Relaxed Lasso	0.7273	0.7220	0.7166	125
(9)	Elastic Net	0.7830	0.7259	0.7190	4,289
