# Supplementary figures and images for: A fast and scalable framework for large-scale and ultrahigh-dimensional sparse regression with application to the UK Biobank
Source: PLoS Genet. 2020 Oct 23;16(10):e1009141. doi: 10.1371/journal.pgen.1009141 (PMC7641476; doi:10.1371/journal.pgen.1009141)

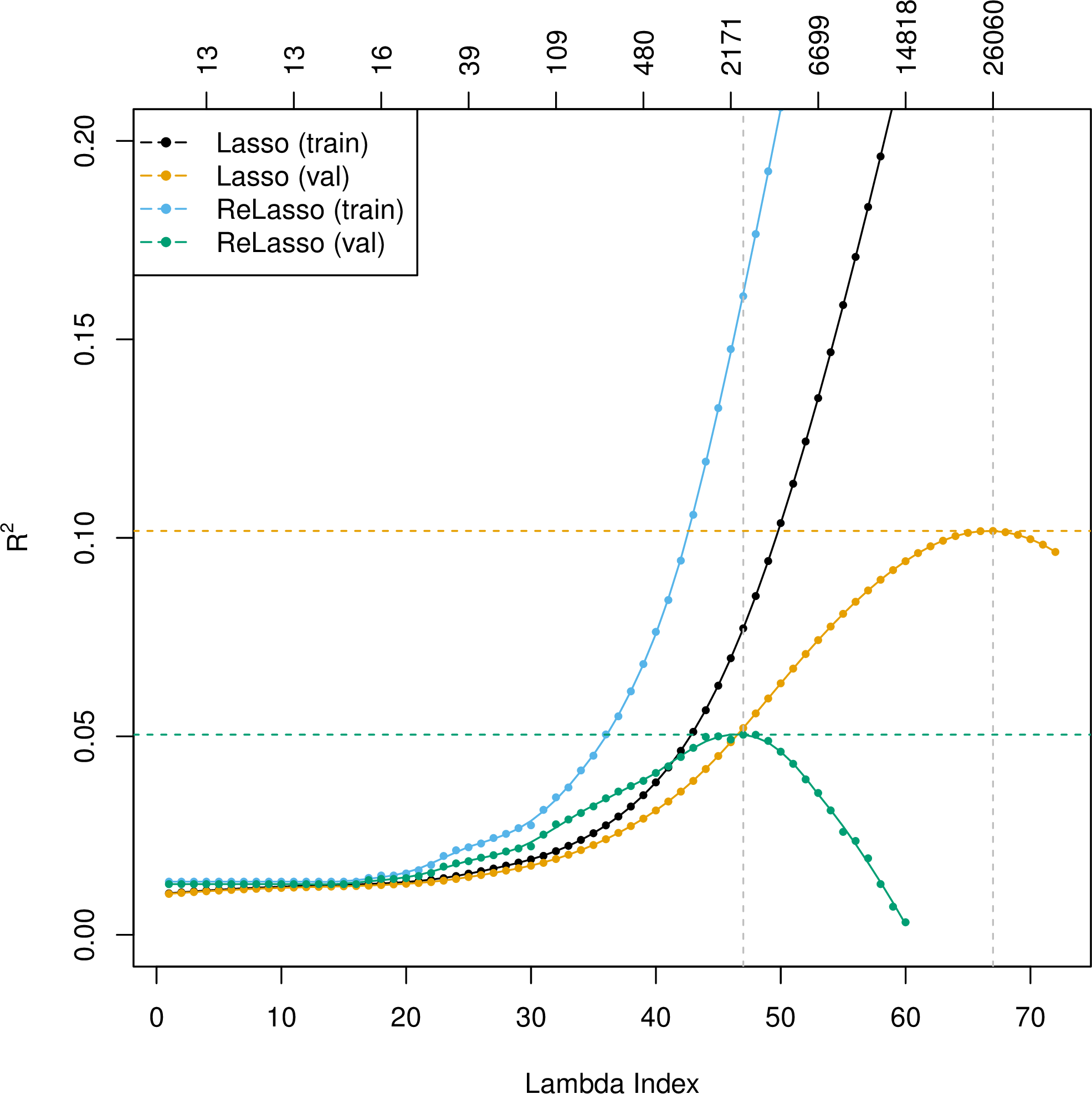

Supplement: S1 Fig — The primary horizontal axis on the bottom represents the index of lambda values, 1 ≤ ℓ ≤ L, which correspond to the sequence of the regularization parameters, λ1 > λ2 > ⋯ > λL. The top axis shows the number of active variables in the model. ReLasso: relaxed lasso. (TIF) [file pgen.1009141.s011.tif]

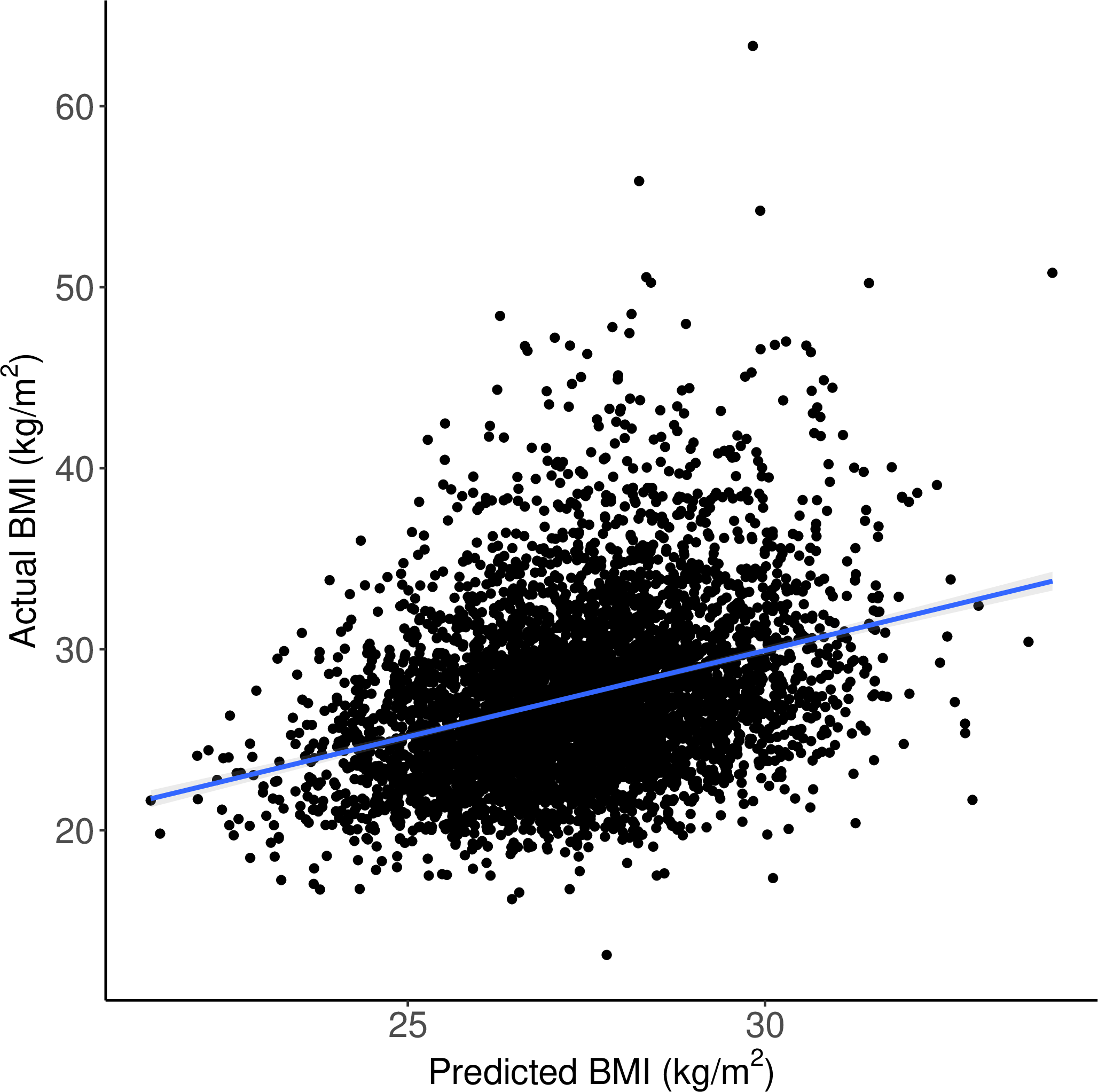

Supplement: S2 Fig — A regression line with its 95% confidence band is also added on top of the dots. The correlation between actual BMI and predicted BMI is 0.3256. (TIF) [file pgen.1009141.s012.tif]

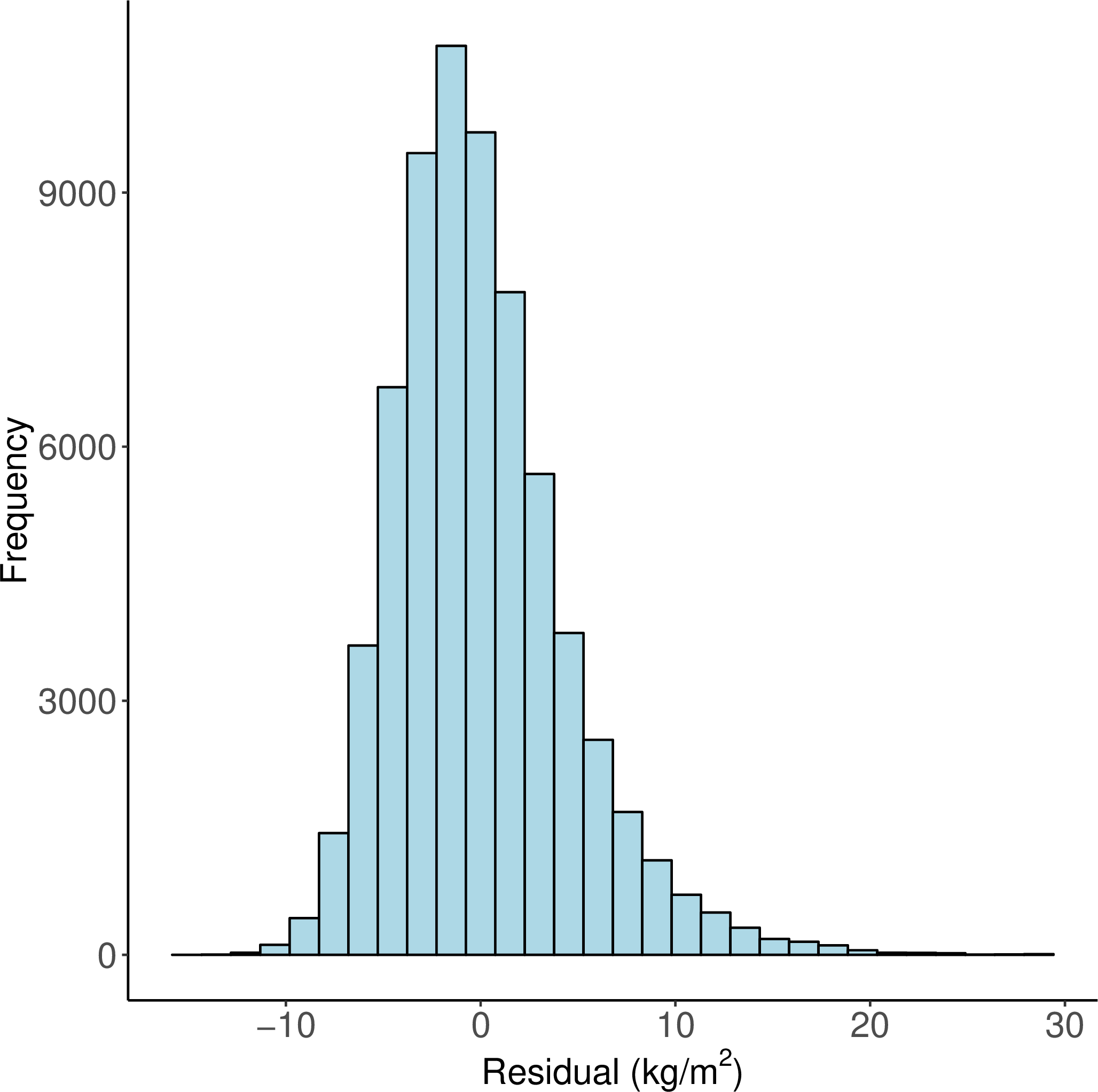

Supplement: S3 Fig — Standard deviation of the residual is 4.51 kg/m2. (TIF) [file pgen.1009141.s013.tif]

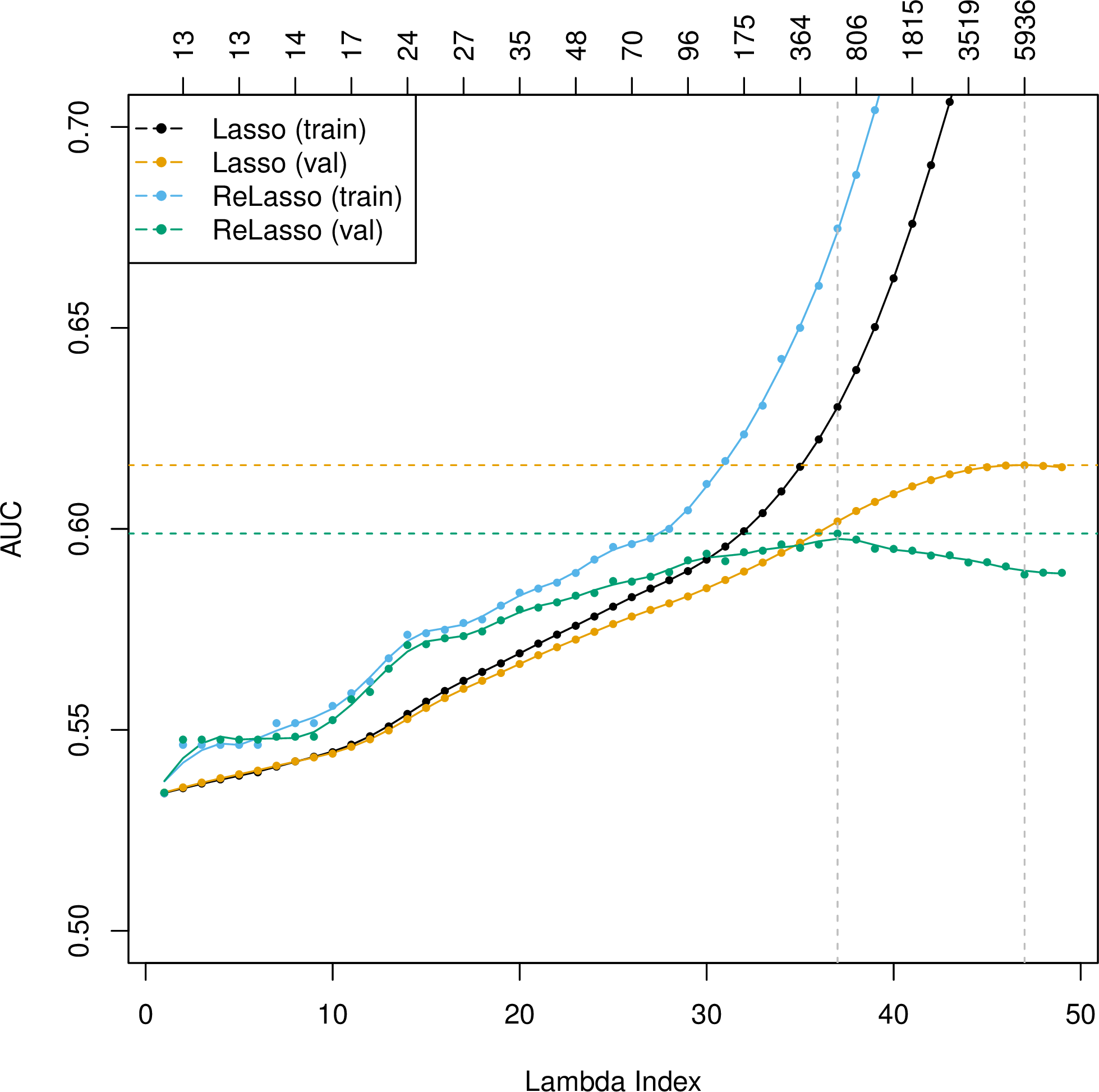

Supplement: S4 Fig — The primary horizontal axis on the bottom represents the index of lambda values, 1 ≤ ℓ ≤ L, which correspond to the sequence of the regularization parameters, λ1 > λ2 > ⋯ > λL. The top axis shows the number of active variables in the model. ReLasso: relaxed lasso. (TIF) [file pgen.1009141.s014.tif]

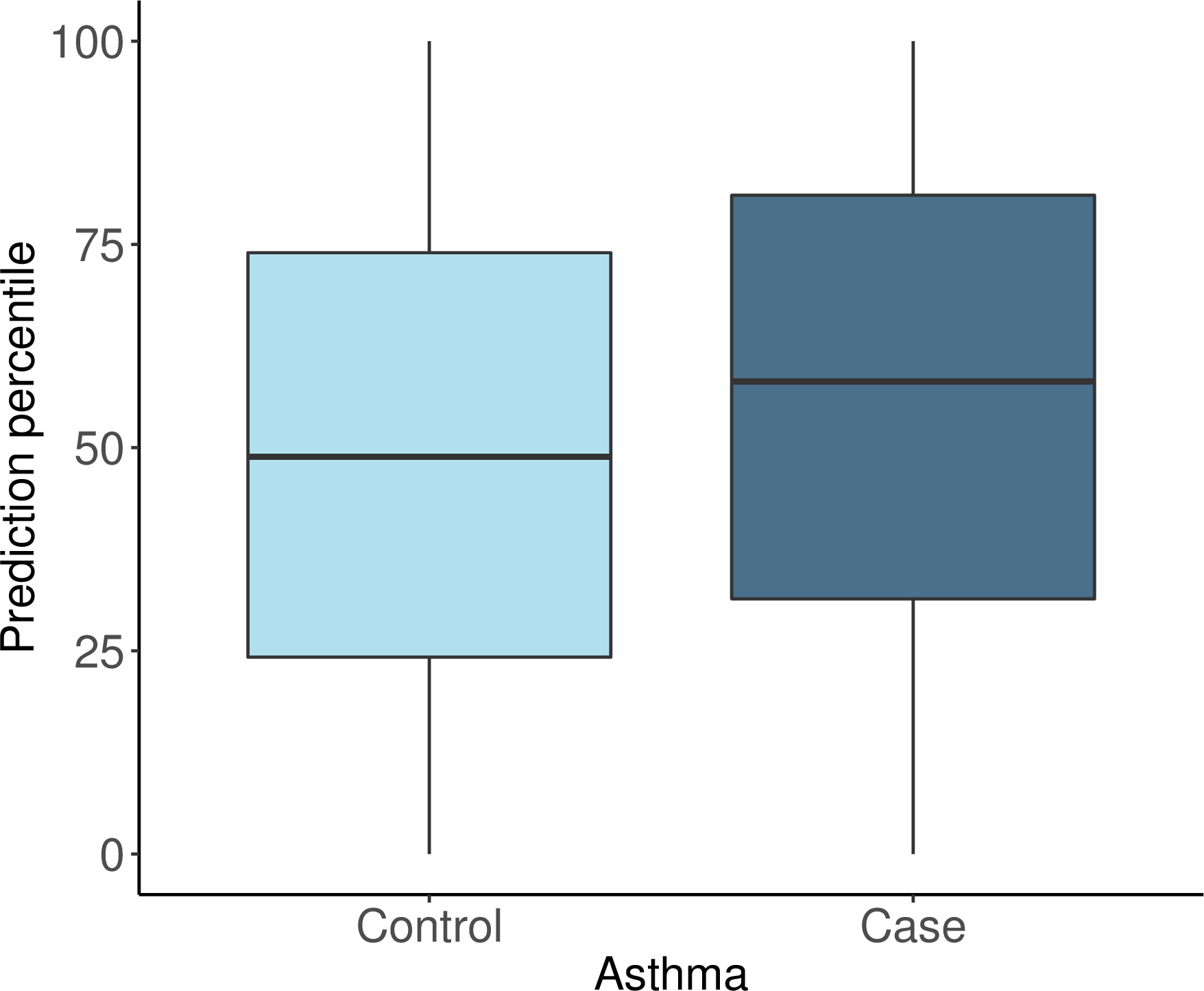

Supplement: S5 Fig — This is based on the optimal lasso model. (TIF) [file pgen.1009141.s015.tif]

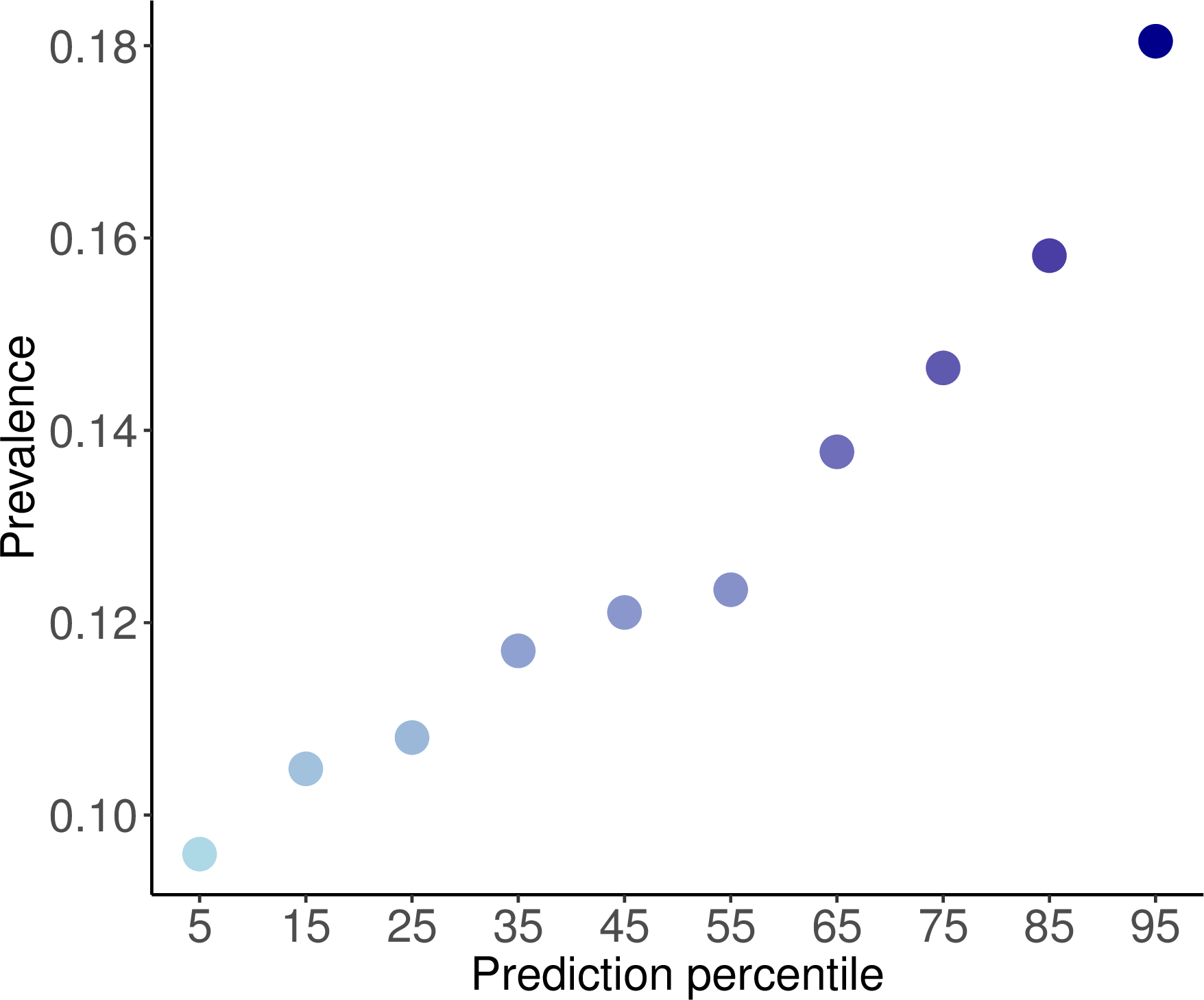

Supplement: S6 Fig — This is based on the optimal lasso model. (TIF) [file pgen.1009141.s016.tif]

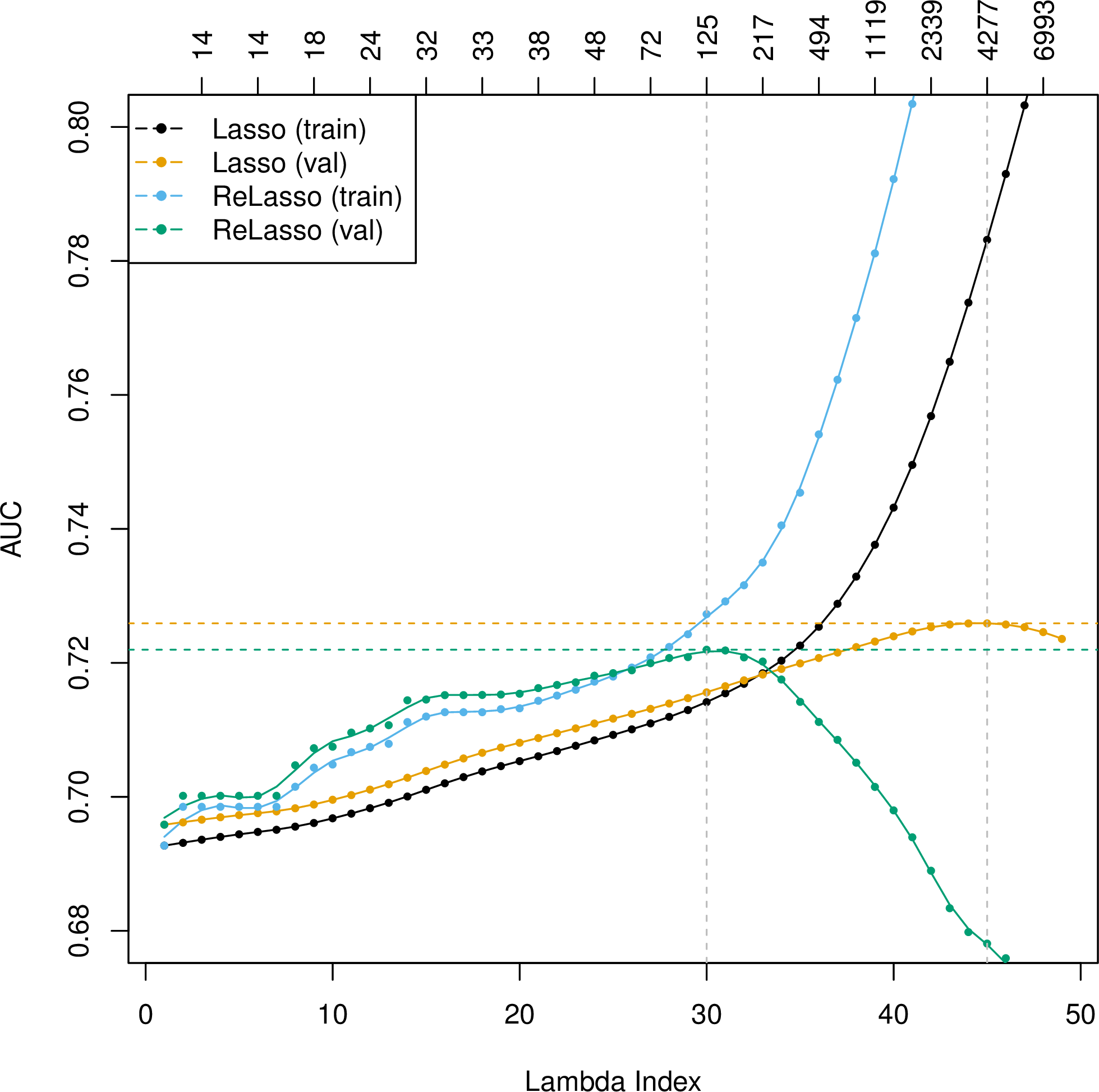

Supplement: S7 Fig — The primary horizontal axis on the bottom represents the index of lambda values, 1 ≤ ℓ ≤ L, which correspond to the sequence of the regularization parameters, λ1 > λ2 > ⋯ > λL. The top axis shows the number of active variables in the model. ReLasso: relaxed lasso. (TIF) [file pgen.1009141.s017.tif]

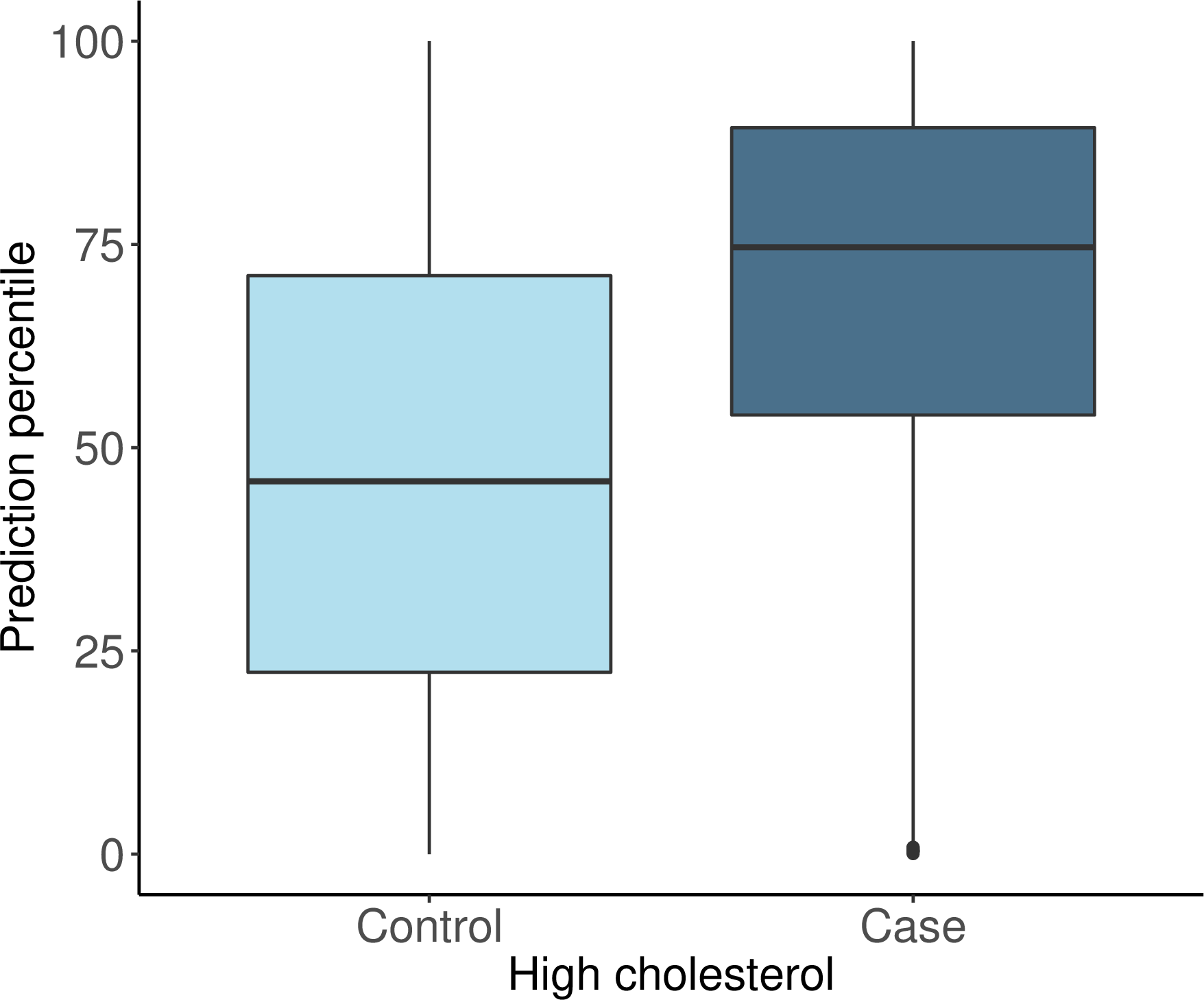

Supplement: S8 Fig — This is based on the optimal lasso model. (TIF) [file pgen.1009141.s018.tif]

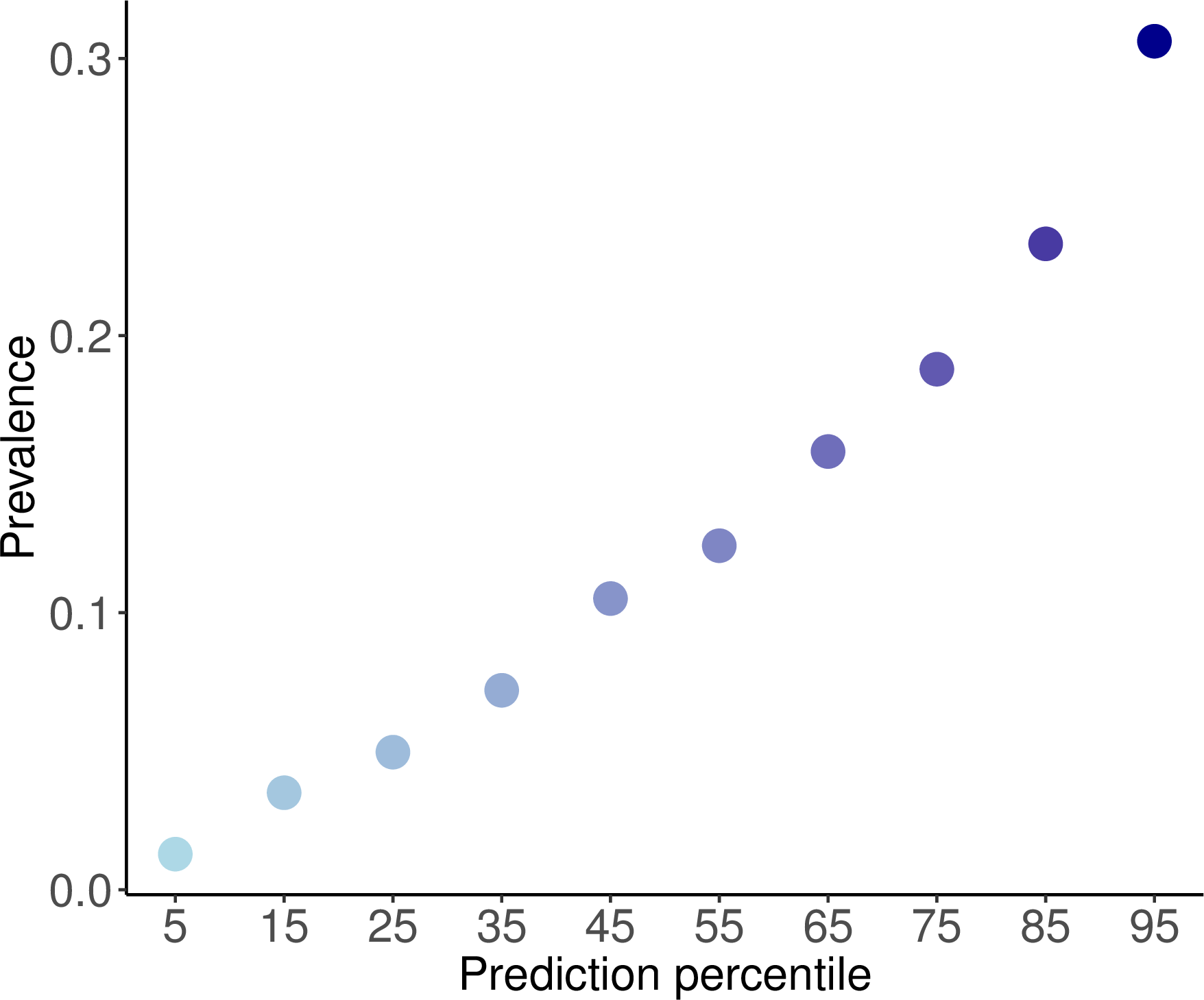

Supplement: S9 Fig — This is based on the optimal lasso model. (TIF) [file pgen.1009141.s019.tif]

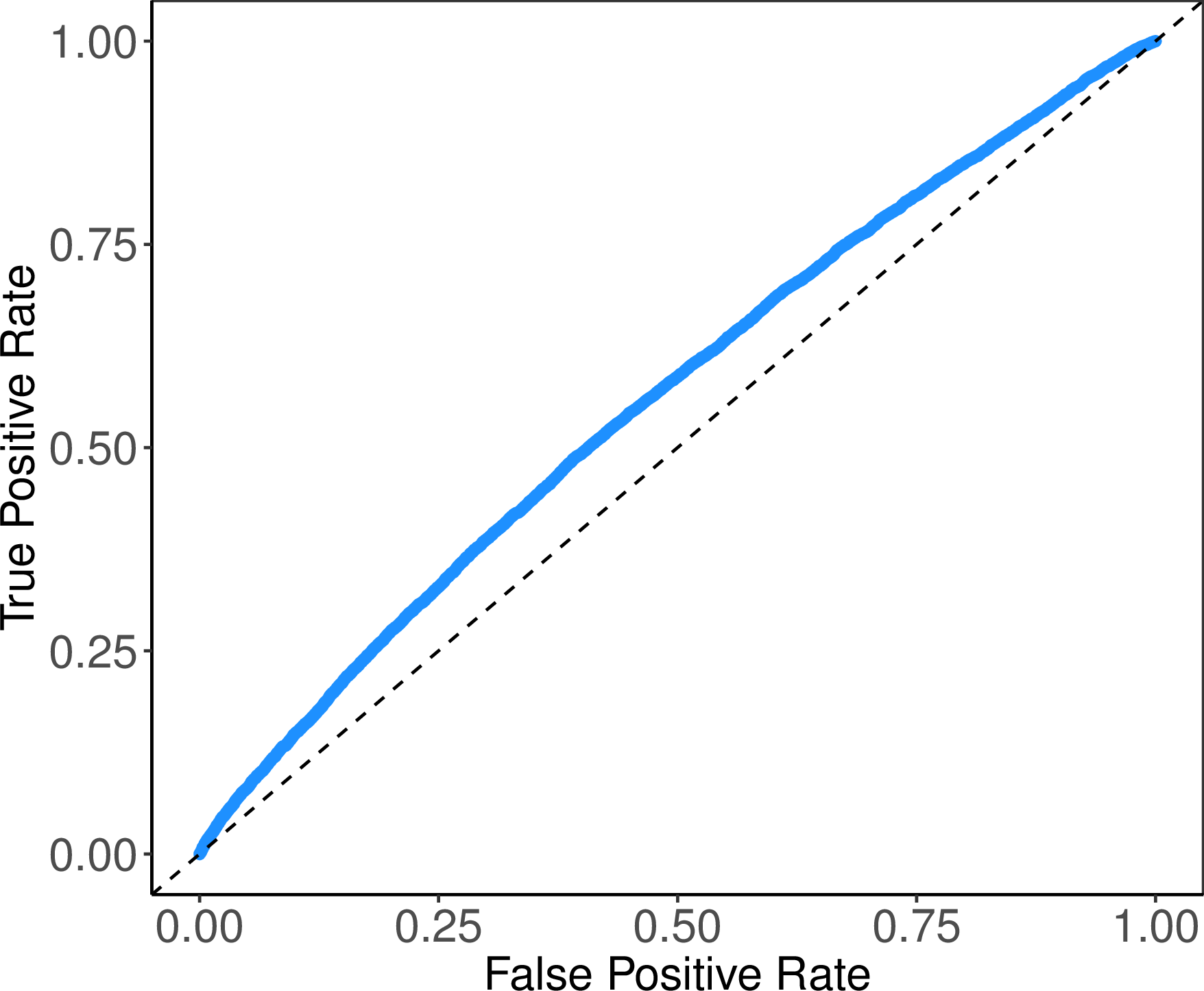

Supplement: S10 Fig — This is based on the optimal lasso model. (TIF) [file pgen.1009141.s020.tif]

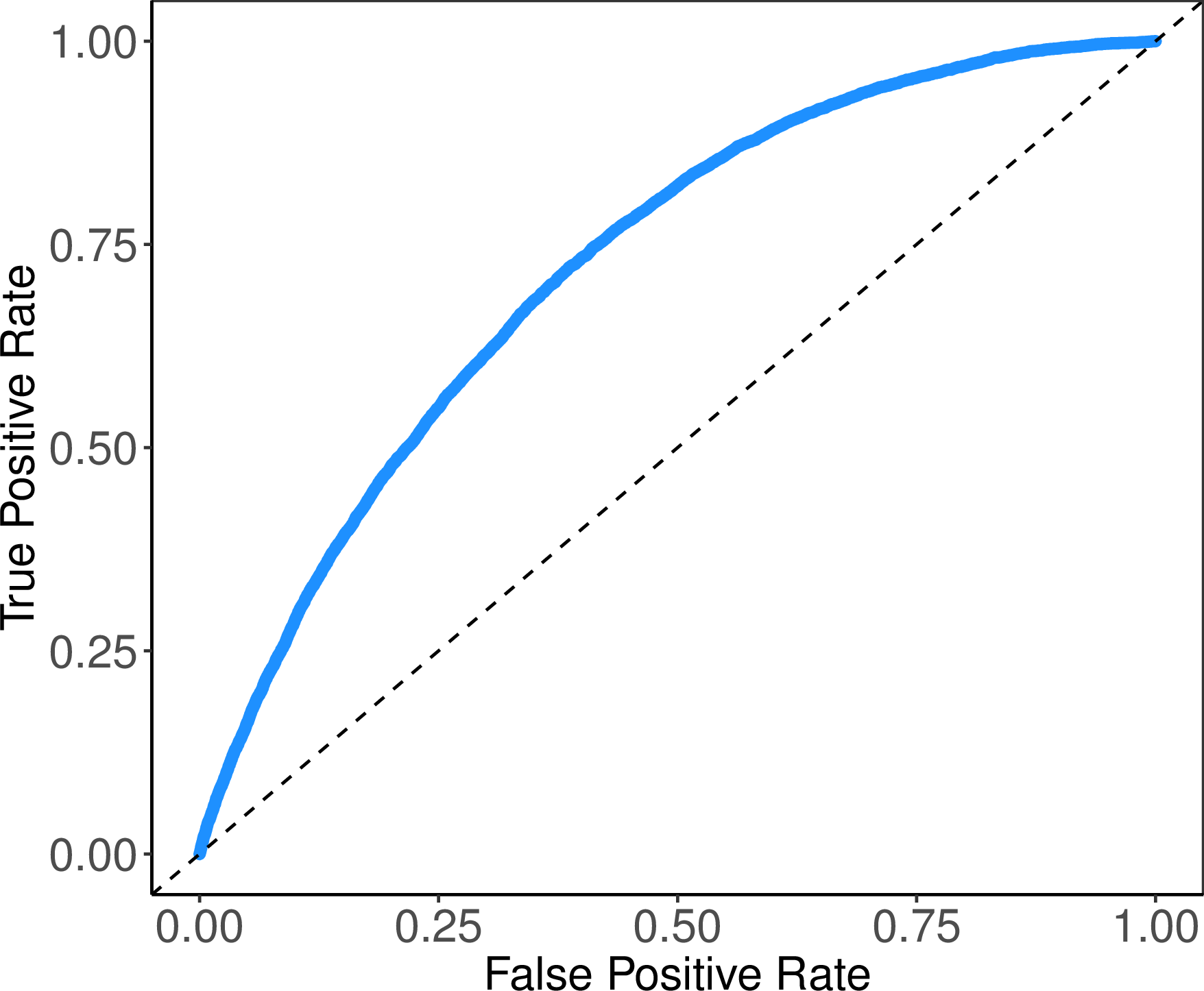

Supplement: S11 Fig — This is based on the optimal lasso model. (TIF) [file pgen.1009141.s021.tif]

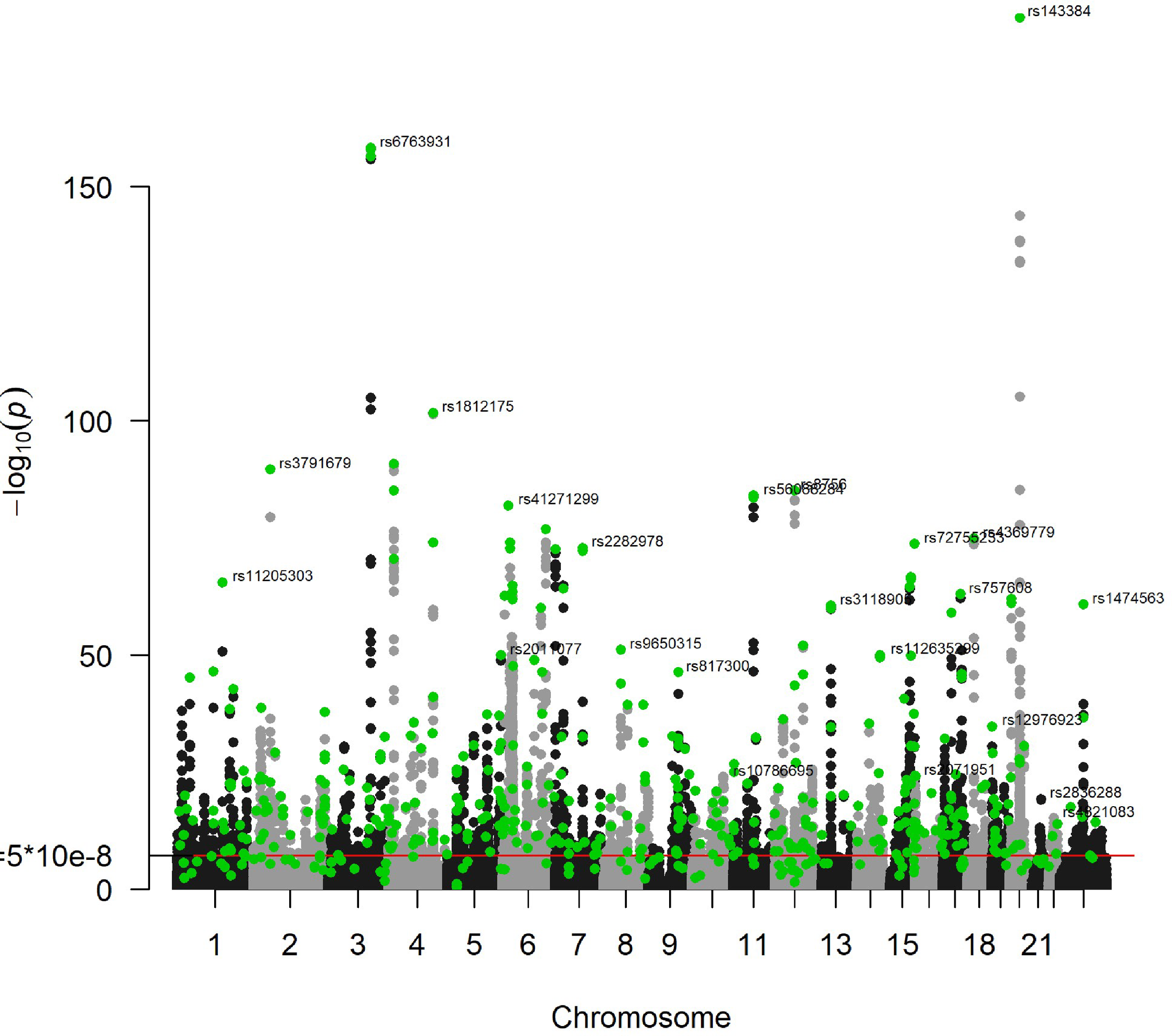

Supplement: S12 Fig — This is based on the optimal lasso model. The vertical axis shows −log10(p) for each SNP. The red horizontal line represents a reference level of p = 5 × 10^−8^. (TIF) [file pgen.1009141.s022.tif]

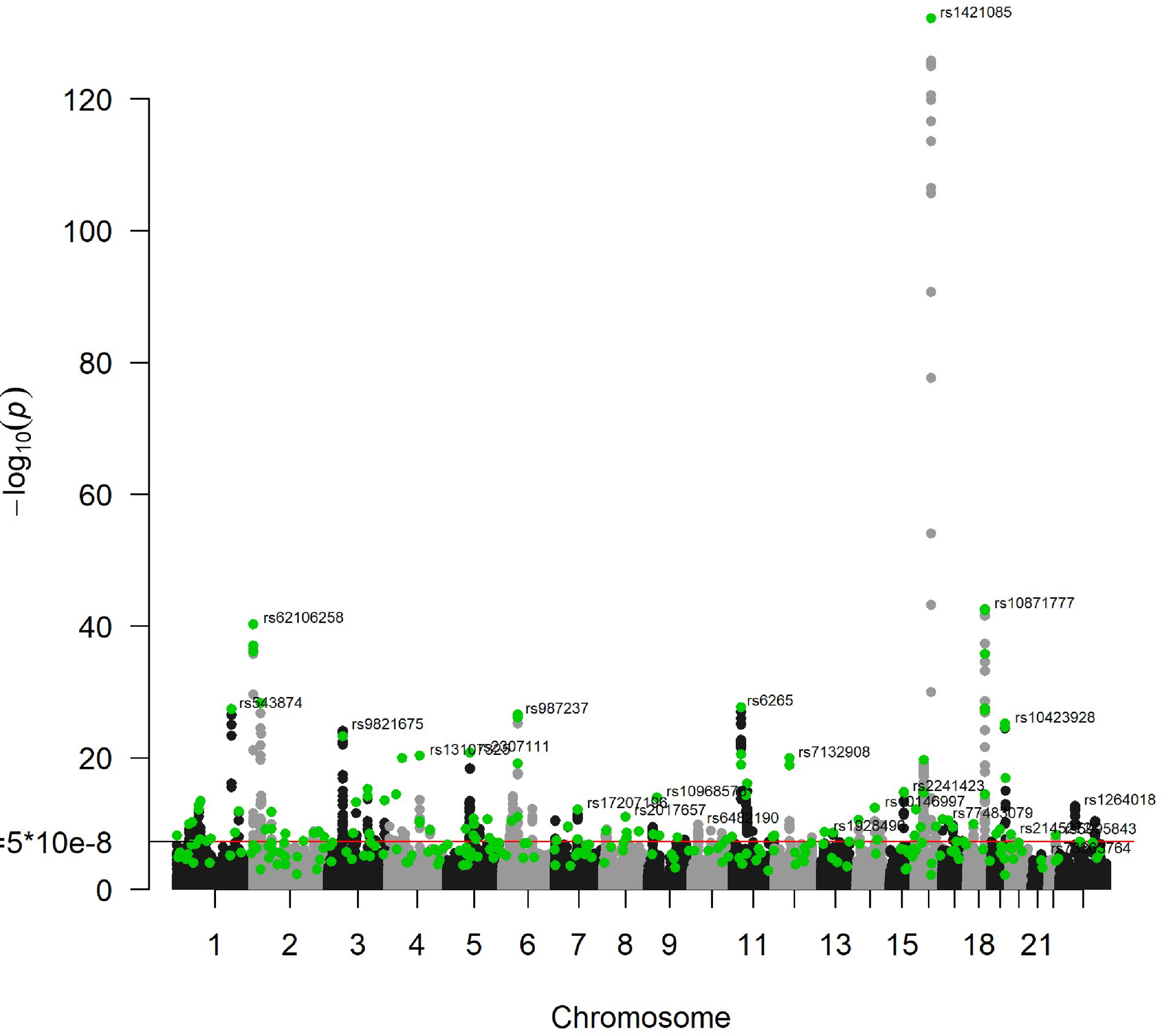

Supplement: S13 Fig — This is based on the optimal lasso model. The vertical axis shows −log10(p) for each SNP. The red horizontal line represents a reference level of p = 5 × 10^−8^. (TIF) [file pgen.1009141.s023.tif]

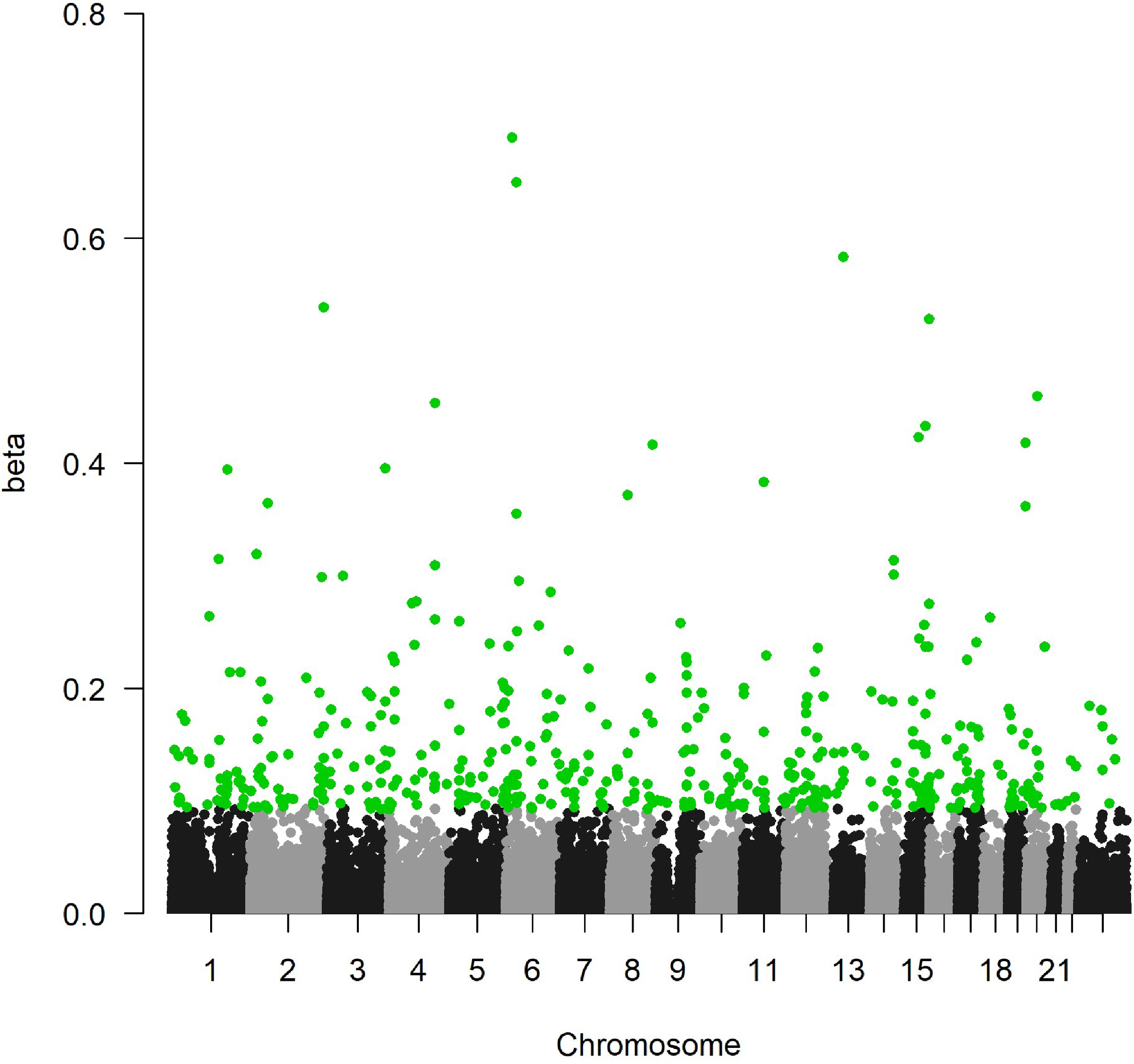

Supplement: S14 Fig — This is based on the optimal lasso model. The vertical axis shows the magnitude of the coefficients from snpnet. The SNPs with relatively large lasso coefficients are highlighted in green. (TIF) [file pgen.1009141.s024.tif]

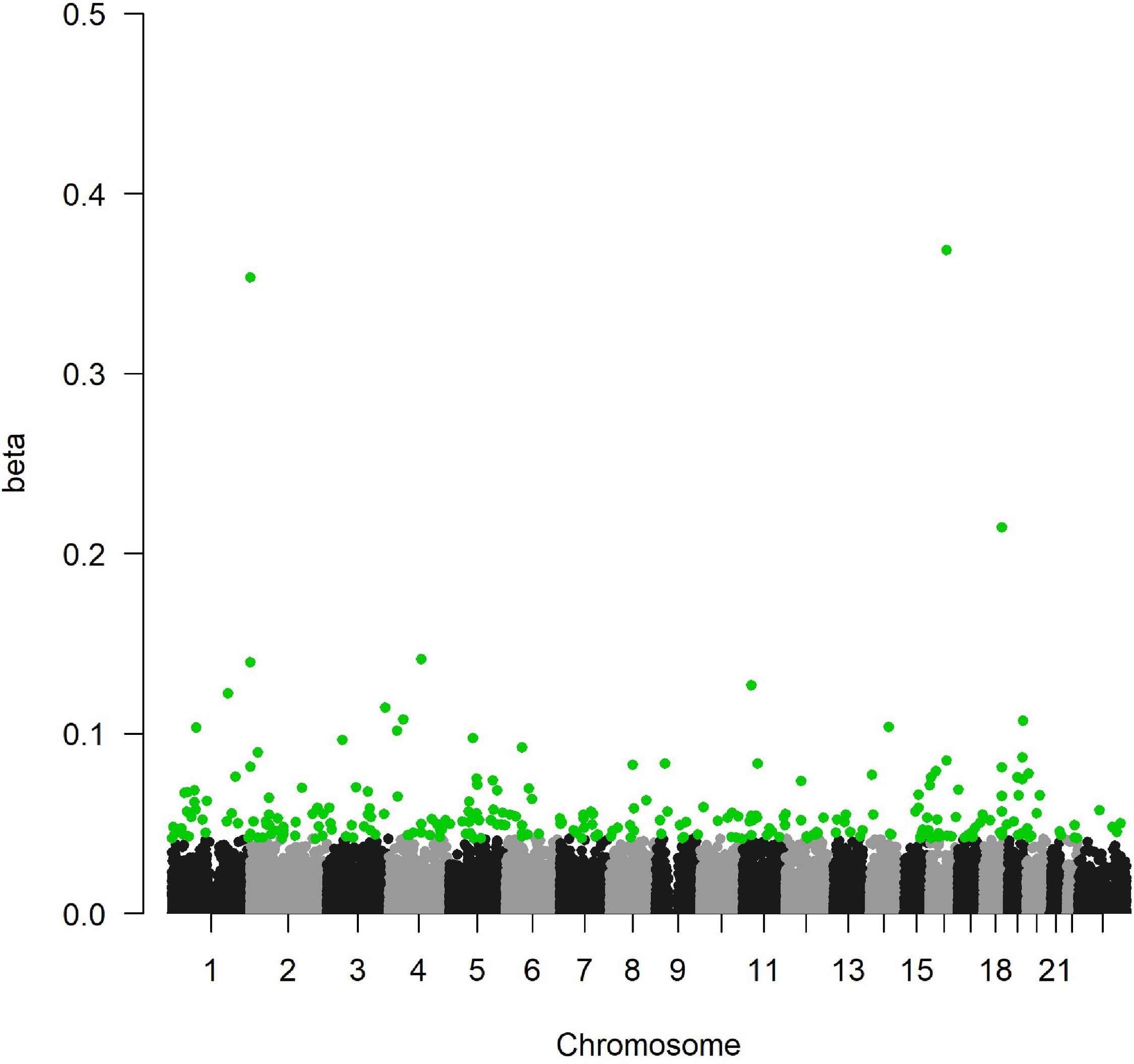

Supplement: S15 Fig — This is based on the optimal lasso model. The vertical axis shows the magnitude of the coefficients from snpnet. The SNPs with relatively large lasso coefficients are highlighted in green. (TIF) [file pgen.1009141.s025.tif]

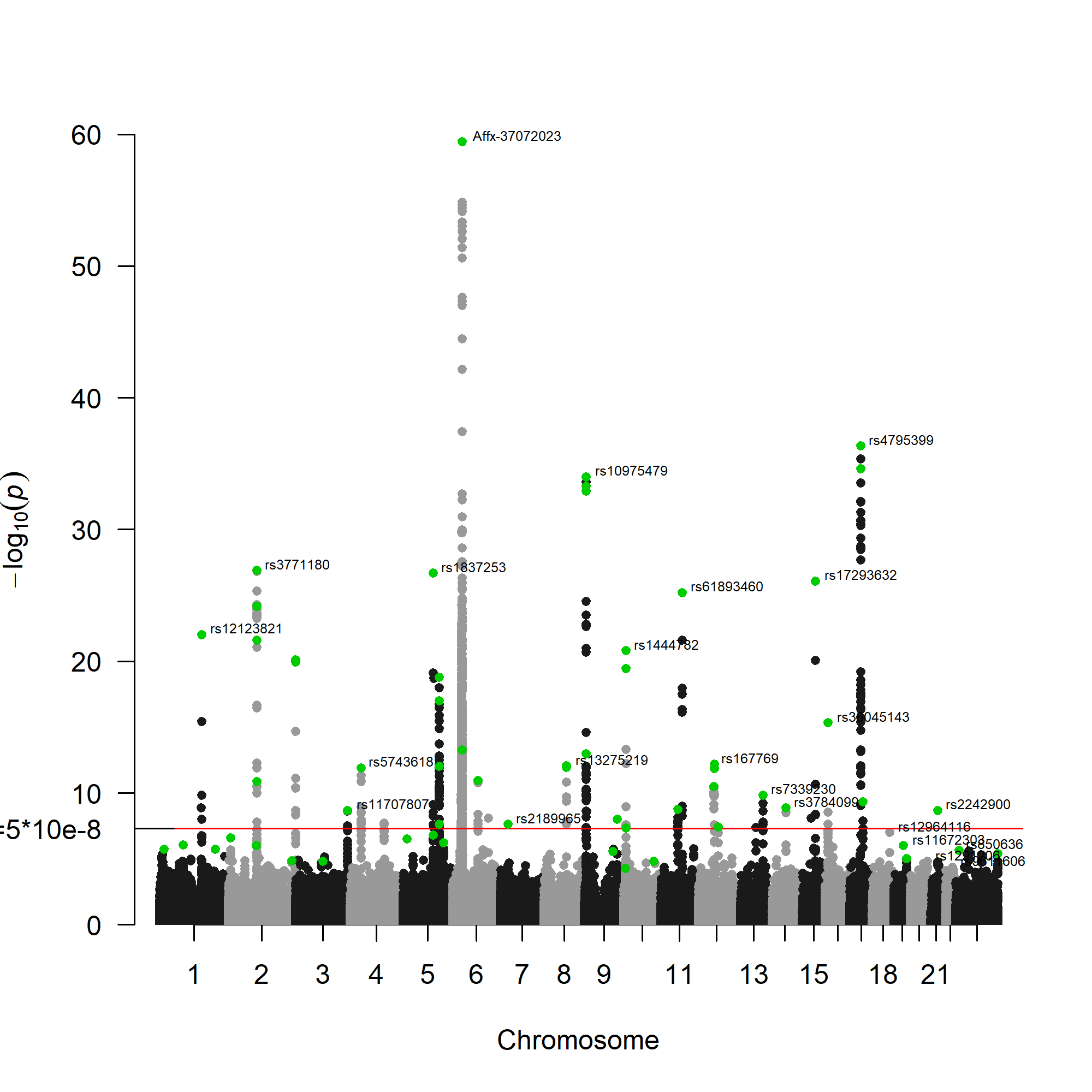

Supplement: S16 Fig — This is based on the optimal lasso model. The vertical axis shows −log10(p) for each SNP. The red horizontal line represents a reference level of p = 5 × 10^−8^. (TIF) [file pgen.1009141.s026.tif]

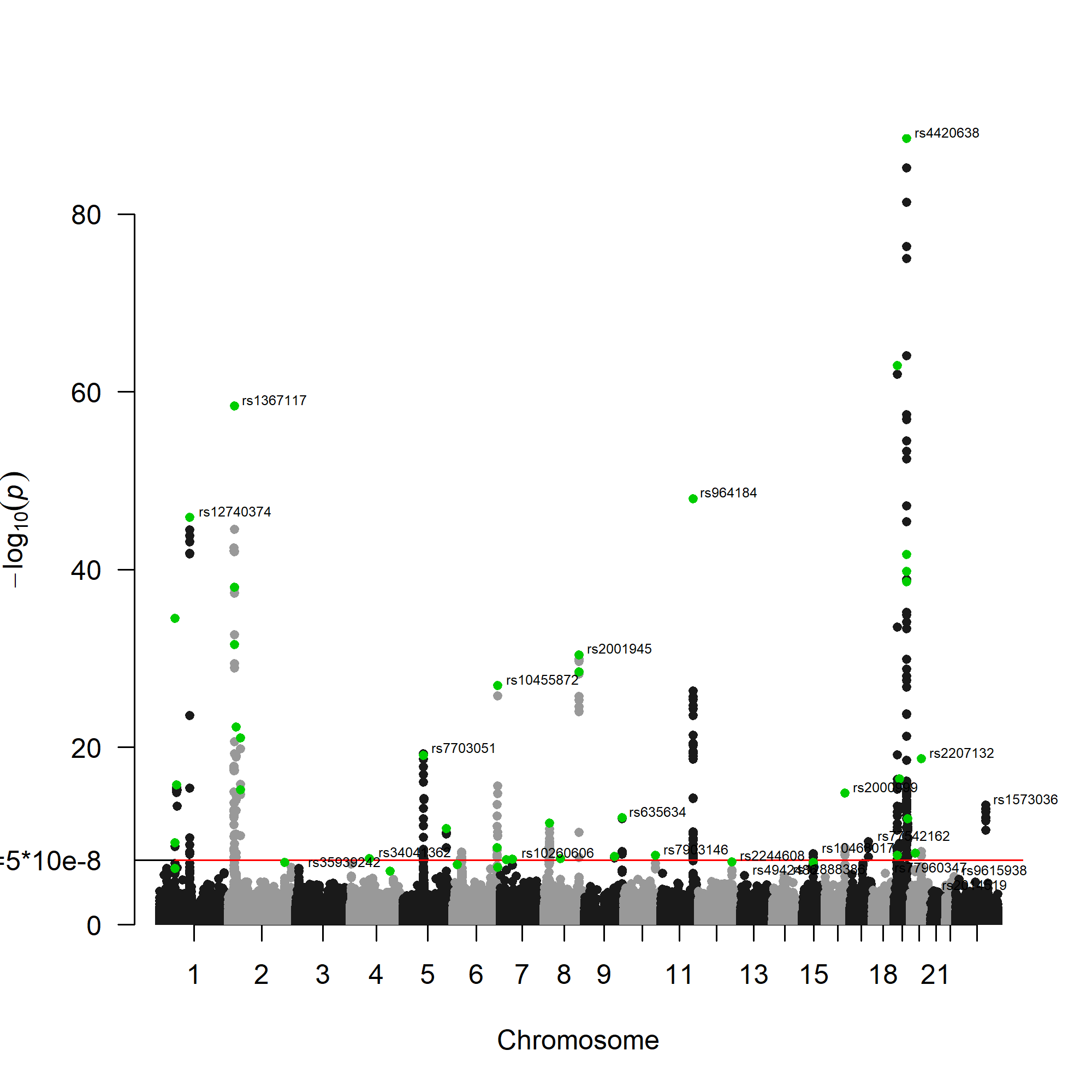

Supplement: S17 Fig — This is based on the optimal lasso model. The vertical axis shows −log10(p) for each SNP. The red horizontal line represents a reference level of p = 5 × 10^−8^. (TIF) [file pgen.1009141.s027.tif]

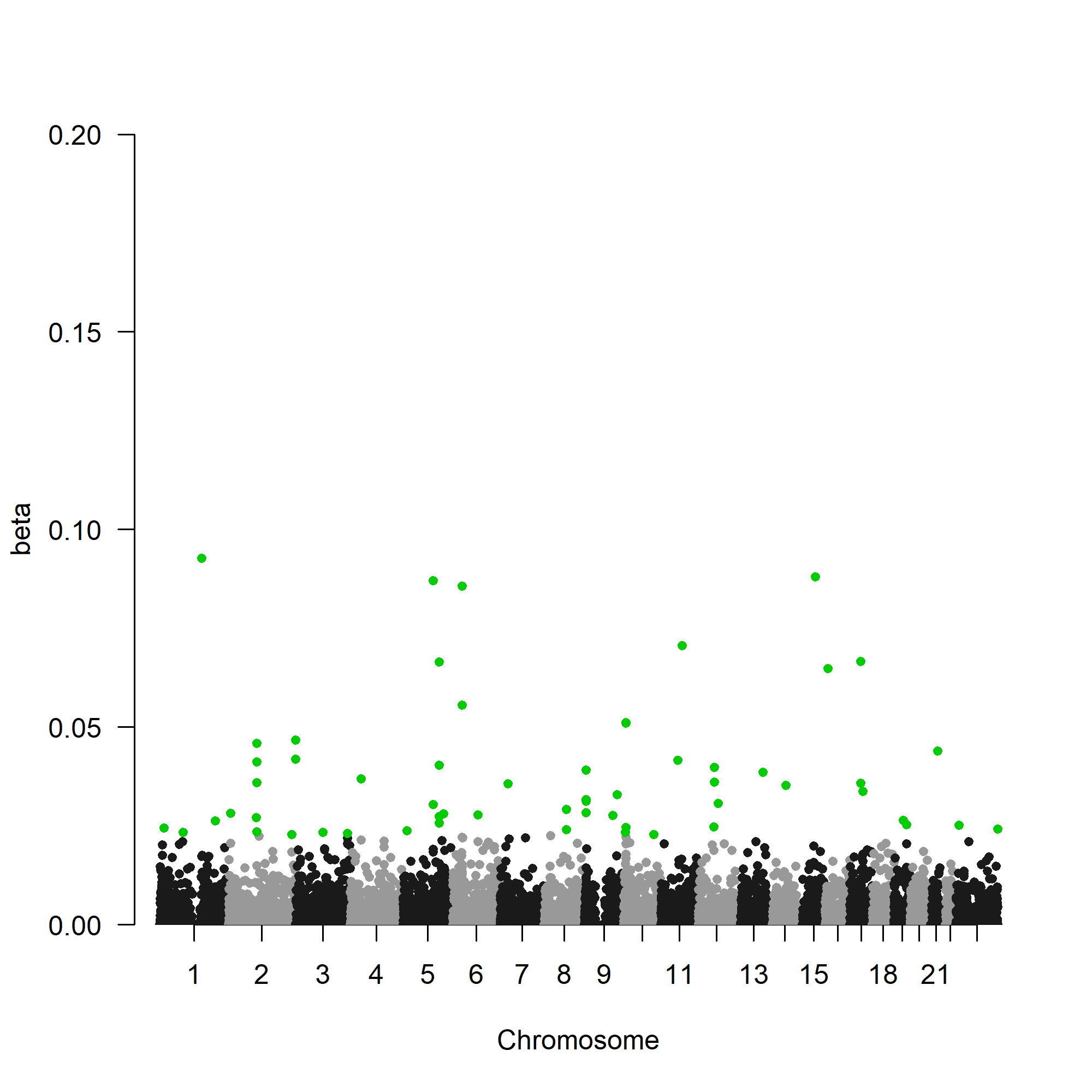

Supplement: S18 Fig — This is based on the optimal lasso model. The vertical axis shows the magnitude of the coefficients from snpnet. The SNPs with relatively large lasso coefficients are highlighted in green. (TIF) [file pgen.1009141.s028.tif]

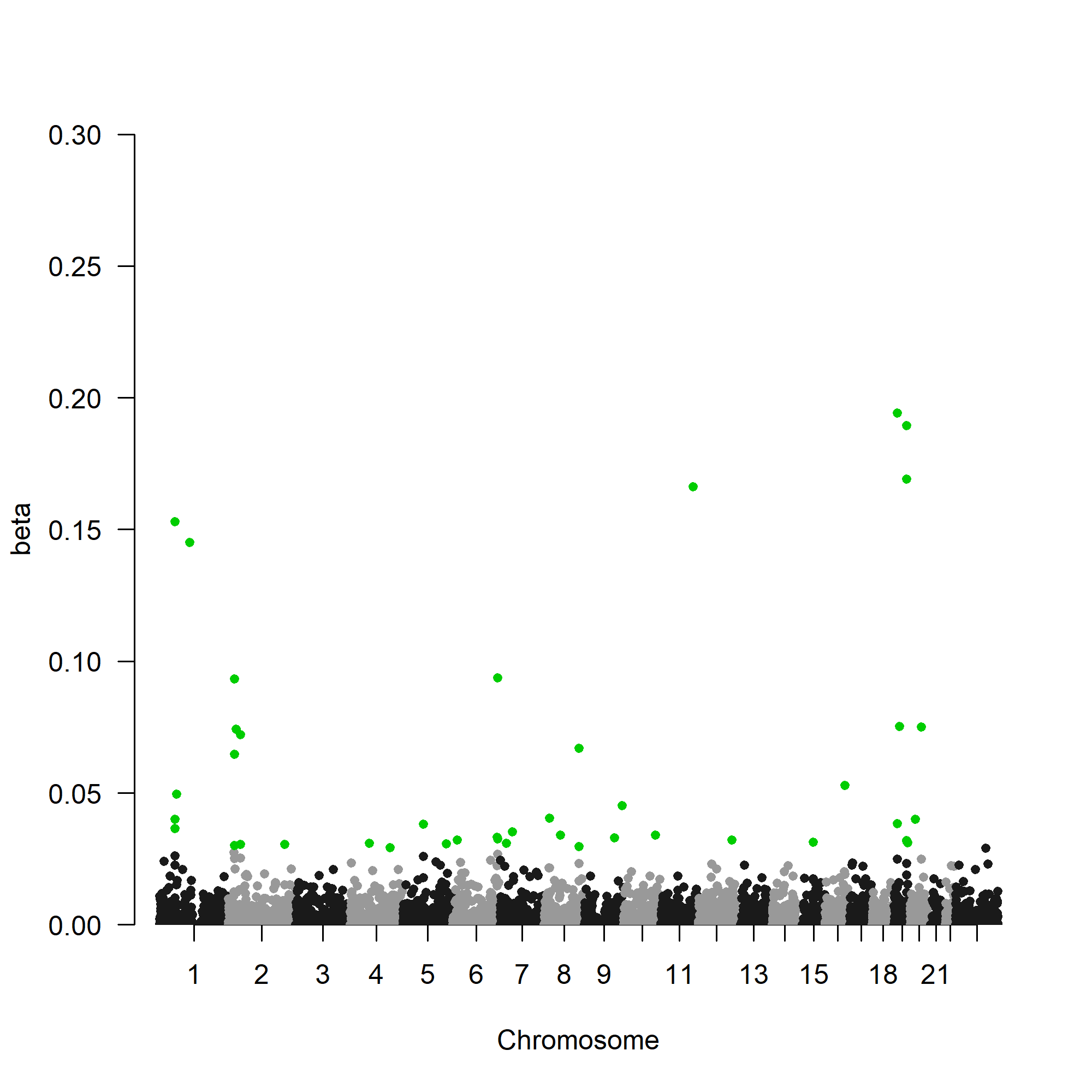

Supplement: S19 Fig — This is based on the optimal lasso model. The vertical axis shows the magnitude of the coefficients from snpnet. The SNPs with relatively large lasso coefficients are highlighted in green. (TIF) [file pgen.1009141.s029.tif]
