# Supplementary material for: A fast and scalable framework for large-scale and ultrahigh-dimensional sparse regression with application to the UK Biobank
Source: PLoS Genet. 2020 Oct 23;16(10):e1009141. doi: 10.1371/journal.pgen.1009141 (PMC7641476; doi:10.1371/journal.pgen.1009141)
Supplement: Attachment — Submitted filename: response_to_reviewers.pdf [file pgen.1009141.s030.pdf]

Dear Dr. Zhu,

Please find the review response and revision regarding our manuscript “A Fast and Scalable Framework for Large-scale and Ultrahigh-dimensional Multivariate Genome-wide Predictive Modeling with Application to the UK Biobank” (PGENETICS-D-20-00068). We thank the reviewers for their constructive comments and their time. We believe that the changes made in the light of their comments have significantly improved the manuscript.

Our responses to the reviewers below are in blue font, the comments from the reviewer are copied in black, and quoted texts from the updated manuscript are shown in gray with a vertical bar (examples are shown below):

This is an example of reviewer’s comments
This is an example of our response.
> This is an example of quoted texts from the updated manuscript

Reviewer #1: Review of Qian et al

Summary:

This paper describes an efficient algorithm for fitting the lasso regression model to large data sets, along with an implementation (R package snpnet) and application to the UK biobank data to obtain predictors (effectively performing genomic prediction, or computing polygenic risk scores, PRS) for several different phenotypes. The paper compares prediction accuracy with some other simpler methods.

The lasso is, in general, a widely studied, and also quite widely used method. As such an algorithm and implementation for very large datasets are of potential interest to a general audience both inside and outside genetics. However, for readers of PloS genetics the interest is going to stand or fall on the application: is Lasso a good method to do genomic prediction? I am skeptical of this: the Lasso has never been the method of choice for genomic prediction in smaller data sets, with the field generally preferring other large-scale regression methods, including very simple methods (eg "ridge regression", usually known as BLUP in the quantitative genetics literature) or very computationally intensive methods (Bayesian regression, usually fit via MCMC). The Elastic Net is also sometimes used. But the Lasso, rarely. While I will keep an open mind on whether this could change for biobank-sized data, the current paper is unconvincing on this because none of the comparisons are with state-of-the-art approaches to this problem.

Overall then I think the main contribution of this paper is the algorithmic ideas, whose main appeal is their simplicity and generality: I like the fact that the design allows the algorithm to maximally exploit previous implementations, rather than having to reimplement the coordinate

ascent steps for example. However, unless the resulting method is really competitive with state of the art for genomic prediction then this seems better suited to another journal.

Thank you very much for taking the time to review the manuscript. We are confident that your comments have improved the quality and clarity of the manuscript.

You have raised an important point here. We apologize that we did not explain the motivation clearly and missed some common methods used in the field. In fact, the BASIL framework can be easily extended to the elastic-net. We added the extension to the package implementation and the revised manuscript (Line 204-226). Compared with the other methods, this class of regularized regression methods with variable selection has benefits both in statistical performance and computation. For the former, we compare the predictive performance with the methods suggested (elastic-net and two bayesian regression methods, PRS-CS and SBayesR) in the summary plot (Figure 2) and Table 1, 5, 6, 7. It turns out that the lasso is fairly competitive and also needs less variables. For the latter, that allows us to focus the computation on a much smaller subset of variables that are affordable by the memory, which is crucial for biobank-sized data.

Detailed comments:

1. Comparisons with other methods:

The methods used here do not seem to represent a reasonable selection of state-of-the-art approaches to forming predictors for genetic data, on which there is a large literature. Historically genomic prediction has been done using multiple linear regression fit either using very simple methods (eg "ridge regression", usually known as BLUP in the quantitative genetics literature) or very computationally intensive methods (Bayesian regression, usually fit via MCMC). More recently, motivated by the difficulty of accessing/sharing genotype data, as well as computational considerations, a literature has sprung up around methods that attempt to build predictors based on summary statistics only (and LD from a reference panel). For example, Ge et al and Lloyd-Jones et al:

https://www.nature.com/articles/s41467-019-09718-5
https://www.nature.com/articles/s41467-019-12653-0

are recent examples, and includes comparisons with other methods, the latter specifically on the UK biobank data with some of the same phenotypes considered here.

To take a quick example, in Fig 2 of Lloyd Jones, looking at R2 for BMI, the performance among the methods they consider ranges from 0.1 to 0.126. In this paper (Table 3) Lasso achieves 0.103. I realize these numbers are not directly comparable, being based on different protocols (CV splits etc) for analyzing the UK biobank data, but it illustrates my concern that Lasso may not be competitive with the best existing methods.

Thank you for pointing us to the recent literature and representative work in the field of genomic prediction. To make fair comparison, we run two bayesian methods (PRS-CS and SBayesR) on our partition of the dataset and summarize the test results in Figure 2 and separately in the results tables for each of the phenotypes. It turns out the lasso is fairly competitive for all the four phenotypes.

It is possible that BLUP/ridge regression does a good job, but it can be computationally expensive unless with specific implementation (that works directly with the compressed data). Nevertheless, we did use elastic-net with small lasso component (alpha = 0.1) as an approximation and evaluate its performance on the test set (after selection of lambda on the validation set):

	Elastic-net (alpha = 0.1)	Lasso (alpha = 1.0)
Height (R^2)	0.6997	0.6999
BMI (R^2)	0.1071	0.1052
Asthma (AUC)	0.6131	0.6126
High Cholesterol (AUC)	0.7180	0.7191

Lasso turns out to be still competitive and selects less variables.

2. Algorithmic description

I found much of the algorithmic description in the overview overly long and hard to follow. The basic idea seems rather simple (which is a good thing!) but the presentation seems to obscure the simplicity rather than highlighting it. The formal presentation of Algorithm 1 in section 4 helps a lot, and I suggest it should be moved to the overview section. This should allow the text in the algorithmic overview to be shortened, since much of the words seems to be repeating, in less precise terms, what is given in Algorithm 1.

Thank you for the suggestion. In the revised manuscript, we moved the algorithm box to the overview section and highlighted the main ingredients. We rewrote the algorithm description in both overview and result sections to make it more succinct.

Also:

- the algorithm and text did not seem to address what happens if the "checking" step fails. That is, in step 5 of Algorithm 1, what if no lambda satisfies the KKT conditions? Or is this guaranteed not to happen?

Thank you for raising this point. It is not guaranteed. We need to expand the strong set and repeat if the checking step all fails. We've corrected this in the presentation of the algorithm (Step 5 in Algorithm 1 and Line 177-182).

- How is M chosen? Does it matter?

Thank you for pointing this out. It implies a tradeoff between the total number of iterations (amount of expensive disk I/Os) and the computational cost of the lasso fitting. We've explained more in the discussion part of the manuscript (Line 361-379).

> In our algorithm, the choice of M is important for the practical performance. It trades off between the number of iterations and the computation per iteration. With a small M or small update of the strong set, it is very likely that we are unable to proceed fast along the lambda sequence in each iteration. Although the design of the BASIL algorithm guarantees that for any M, Delta M > 0, we are able to obtain the full solution path after sufficient iterations, many iterations will be needed if M is chosen too small, and the disk I/O cost will be dominant. In contrast, a large M will incur more memory burden and more expensive lasso computation, but with the hope to find more valid lasso solutions in one iteration, save the number of iterations and the disk I/O. It is hard to identify the optimal M a priori. It depends on the computing architecture, the size of the problem, the nature of the phenotype, etc. For this reason, we tend to leave it as a subjective parameter to the user's choice. However in the meantime, we do plan to provide a more systematic option to determine M, which leverages the strong rules again. Recall that in the simple setting with no intercept and no covariates, the initial strong set is constructed by |x_j^T y| <= 2*lambda - lambda_{max}. Since the strong rules rarely make mistakes and are fairly effective in discarding inactive variables, we can guide the choice of batch size M by the number of lambda values we want to cover in the first iteration. For example, one may want the strong set to be large enough to solve for the first 10 lambda's in the first iteration. We can then let M = |{1 <= j <= p: |x_j^T y| > 2 lambda_10 - lambda_{max}}|. Despite being adaptive to the data in some sense, this approach is by no means computationally optimal. It is more based on heuristics that the iteration should make reasonable progress along the path.

- the algorithm seems to rely on the fact that marginal screening is going to be effective at identifying the correct variables to add in. In some cases with complex correlations among variables this may not be true - one can construct problems where the best pair of variables to include are not among the marginally strongest. How does the algorithm cope with that kind of situation? Is it guaranteed to converge in practice?

The algorithm guarantees by the KKT check that the solution is exact to the original, full lasso problem. It is possible (depending on the batch size M) that the first screening doesn't include all the variables necessary for the full lasso solution, then it will fail the KKT check and more variables will be added in the second round and so on until the KKT check passes. So it is guaranteed to converge and achieve the exact full solution.

3. Standardization

The question of whether or not to standardize variables is usually phrased in terms of modeling assumptions -- if rare SNPs have bigger effects than common SNPs then standardization could be appropriate and improve predictive performance. The paper suggests that standardization will produce worse performance but this is not obvious a priori - it should be shown empirically.

Thank you for raising this point. However, we did not claim that standardization will produce worse performance, but that it could bring unintended advantages to some variables in the selection result. In fact, standardization is a tricky phenomenon, and should probably not be used as a vehicle for boosting a SNPs performance. All SNPs are measured on the same scale, so reasonable not to standardize. We have a penalty strength parameter which allows one to alter the relative penalty on a SNP by SNP basis. That can be used instead to boost the chances of rare SNPS for inclusion, and can be based on whatever consideration makes sense.

4. Implementation

The software implementation does not appear to be quite ready for widespread distribution (e.g. the R package on github has no man pages, and I could not find a minimal working example).

Thank you very much for checking on this and raising the issue. This is indeed an important part that we have worked on since the previous submission. It now provides documentation as well as a vignette page that shows basic usages.

Some example pages are attached below.

snpnet {snpnet} R Documentation

Fit the Lasso/Elastic-Net for Large Phenotype-Genotype Datasets

Description

Fit the entire lasso or elastic-net solution path using the Batch Screening Iterative Lasso (BASIL) algorithm on large phenotype-genotype datasets.

Usage

```
snpnet(genotype.pfile, phenotype.file, phenotype, family = NULL, covariates = NULL, alpha
  = 1, nlambda = 100, lambda.min.ratio = ifelse(nobs < nvars, 0.01, 1e-04), split.col = NULL,
  p.factor = NULL, status.col = NULL, mem = NULL, configs = NULL)
```

Arguments

`genotype.pfile`	the PLINK 2.0 pgen file that contains genotype. We assume the existence of genotype.pfile.pgen,pvar.zst,psam.
`phenotype.file`	the path of the file that contains the phenotype values and can be read as as a table. There should be FID (family ID) and IID (individual ID) columns containing the identifier for each individual, and the phenotype column(s). (optional) some covariate columns and a column specifying the training/validation split can be included in this file.
`phenotype`	the name of the phenotype. Must be the same as the corresponding column name in the phenotype file.
`family`	the type of the phenotype: "gaussian", "binomial", or "cox". If not provided or NULL, it will be detected based on the number of levels in the response.
`covariates`	a character vector containing the names of the covariates included in the lasso fitting, whose coefficients will not be penalized. The names must exist in the column names of the phenotype file.

Snpnet Vignette

Junyang Qian and Trevor Hastie

2020-05-08

Introduction

Snpnet is a package that is used to fit the lasso on big genomics data. We assume the data are stored in .pgen/.pvar/.psam format by the PLINK library. The potential training/validation split can be specified with a separate column in the phenotype file.

The most essential parameters in the core function `snpnet` include:

- `genotype.pfile`: the PLINK 2.0 pgen file that contains genotype. We assume the existence of genotype.pfile.{pgen,pvar.zst,psam}.
- `phenotype.file`: the path of the file that contains the phenotype values and can be read as a table.
- `phenotype`: the name of the phenotype. Must be the same as the corresponding column name in the phenotype file.
- `covariates`: a character vector containing the names of the covariates included in the lasso fitting, whose coefficients will not be penalized. The names must exist in the column names of the phenotype file.
- `family`: the type of the phenotype: "gaussian", "binomial" or "cox". If not provided or NULL, it will be detected based on the number of levels in the response.
- `alpha`: the elastic-net mixing parameter, where the penalty is defined as $\alpha \cdot \|\beta\|_1 + (1-\alpha) \cdot \|\beta\|_2^2/2$. `alpha = 1` corresponds to the lasso penalty, while `alpha = 0` corresponds to the ridge penalty.
- `split.col`: the column name in the phenotype file that specifies the membership of individuals to the training or the validation set. The individuals marked as `"train"` and `"val"` will be treated as the training and validation set, respectively. When specified, the model performance is evaluated on both the training and the validation sets.
- `status.col`: the column name for the status column for Cox proportional hazards model. When running the Cox model, the specified column must exist in the phenotype file.
- `mem`: Memory (MB) available for the program. It tells PLINK 2.0 the amount of memory it can harness for the computation. IMPORTANT if using a job scheduler.

Comparison with Glmnet

To compare with **glmnet**, we need to convert the genotype data into a normal R object.

```
ids <- readIDsFromPsam(paste0(genotype.pfile, '.psam'))
phe <- readPheMaster(phenotype.file, ids, "gaussian", covariates, phenotype, NULL, NULL,
configs)
vars <- readRDS(system.file("extdata", "vars.rds", package = "snpnet"))
pvar <- pgenlibr::NewPvar(paste0(genotype.pfile, '.pvar.zst'))
pgen <- pgenlibr::NewPgen(paste0(genotype.pfile, '.pgen'), pvar = pvar, sample_subset =
NULL)
data.X <- pgenlibr::ReadList(pgen, seq_along(vars), meanimpute=F)
colnames(data.X) <- vars
p <- ncol(data.X)
pnas <- numeric(p)
for (j in 1:p) {
  pnas[j] <- mean(is.na(data.X[, j]))
  data.X[is.na(data.X[, j]), j] <- mean(data.X[, j], na.rm = T)  # mean imputation
}
```

```
data.X <- as.matrix(cbind(age = phe$age, sex = phe$sex, phe[, paste("PC", 1:10, sep =
"")], data.X))
data.y <- phe$QPHE
pfactor <- rep(1, p + 12)
pfactor[1:12] <- 0  # we don't penalize the covariates

fit_glmnet <- glmnet::glmnet(data.X, data.y, penalty.factor = pfactor, standardize = F)
```

Other

- the use of the term "multivariate" in the context of a multiple regression with univariate outcome is rather confusing. From the title I expected the paper to deal with multivariate outcomes. Better to stick to "multiple regression", or perhaps "multi-SNP regression" if you prefer.

Thank you for this suggestion. We've taken special care of this in the revised manuscript.

- references to heritability were also confusing. E.g. the abstract refers to "state-of-the-art heritability estimation", when the goal here seems not to be heritability estimation but building a predictor, which are different things. Heritability provides an upper bound on prediction accuracy from genetic data, but building a predictor is not the same as "estimating heritability", and most approaches to estimating heritability do not explicitly build predictors. I think you can (and probably should) write the whole paper without mentioning heritability, and focussing entirely on PRS and prediction accuracy.

Thank you for this suggestion. We've removed all our method's references to heritability estimation and only left some mentioning of heritability in the comparison part that refers specifically to the upper bound of explained variance.

The presentation of result is much longer than it need be. The main results for different phenotypes and methods could probably be shown in a single figure (e.g. Lloyd-Jones Fig 2). Many of the other figures did not seem essential to the main story.

Thank you for pointing this out and the suggestion. In the revised manuscript, we put on a summary plot for the test result comparison (Figure 2 in the manuscript and also as below), focus on the discussion of height in the main test and move details about the other three phenotypes to the appendix.

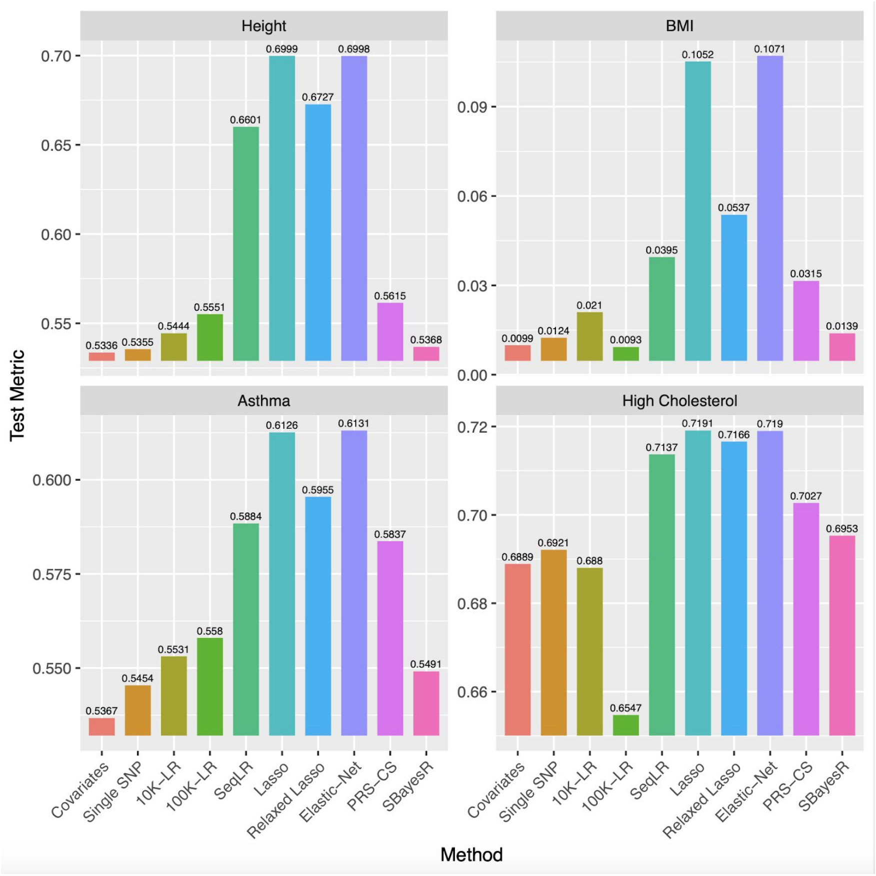

Refs:

Ge, T., Chen, C., Ni, Y. et al. Polygenic prediction via Bayesian regression and continuous shrinkage priors. Nat Commun 10, 1776 (2019). https://doi.org/10.1038/s41467-019-09718-5

Lloyd-Jones, L.R., Zeng, J., Sidorenko, J. et al. Improved polygenic prediction by Bayesian multiple regression on summary statistics. Nat Commun 10, 5086 (2019). https://doi.org/10.1038/s41467-019-12653-0

Reviewer #2: How to build the best predictive model using large-scale genetic data is important in health and disease studies. This paper provides a true regression approach for this problem, an important alternative to the polygenic risk scores. The results from analysis of the UK Biobank are convincing and interesting. The algorithm seems to be quite reasonable.

Thank you very much for taking the time to review the manuscript. Your comments help us fill in some blanks we missed out in the earlier version.

I only have a few minor comments - (1) since Lasso results in biased estimates of the regression coefficients. Do the authors think that by performing further debased estimation, one can further improve the prediction performance? (2) since a very large number of SNPs are selection for each of the data examples, would the consistency results still hold? Lasso theory requires that the model has to be very sparse. (3) Why univariate screening + Lasso does not perform as well as fitting Lasso using all the SNPs? Does this mean that the univariate screening as proposed by Jianqin Fan etc does not really work in the settings considered in this paper?

Thank you for the comments. (1) In fact, we have performed a type of debiased estimation, the relaxed lasso in the comparison. See predictive performance in Figure 2, Table 1, 5, 6, 7 and the solution path in Figure 3, 7, 9, 11 It turns out that the method started to overfit much earlier on the path and did not end up achieving as good performance as the lasso/elastic-net does. (2) We made comments on this in the discussion section of the revised manuscript (Line 394-402).

The lasso has nice variable selection and prediction properties if the linear model assumption together with some additional assumptions such as the restricted eigenvalue condition (Bickel et al., 2009) or the irrepresentable condition (Zhao and Yu, 2006) holds. In practice, such assumptions do not always hold and are often hard to verify. In our UK Biobank application, we don't attempt to verify the exact conditions, and the selected model can be subject to false positives. However, we demonstrate relevance of the selection via empirical consistency with the GWAS results. We have seen superior prediction performance by the lasso as a regularized regression method compared to other methods. More importantly, by leveraging the sparsity property of the lasso, we are able to manage the ultrahigh-dimensional problem and obtain a computationally efficient solution.

(3) This is analogous to the proposal by Lello, et al. (2018) and we made a detailed comparison for height (Line 334-343 and Figure 5).
