# Supplementary material for: A fast and scalable framework for large-scale and ultrahigh-dimensional sparse regression with application to the UK Biobank
Source: PLoS Genet. 2020 Oct 23;16(10):e1009141. doi: 10.1371/journal.pgen.1009141 (PMC7641476; doi:10.1371/journal.pgen.1009141)
Supplement: Attachment — Submitted filename: response_to_reviewers_v2.pdf [file pgen.1009141.s031.pdf]

Dear Dr. Zhu,

Please find the review response and revision regarding our manuscript "**A Fast and Scalable Framework for Large-scale and Ultrahigh-dimensional Sparse Regression with Application to the UK Biobank**" (PGENETICS-D-20-00068R1). We thank the reviewers for their constructive comments and their time. We believe that the changes made in the light of their comments have significantly improved the manuscript.

Our responses to the reviewers below are in blue font, the comments from the reviewer are copied in black, and quoted texts from the updated manuscript are shown in gray with a vertical bar (examples are shown below):

This is an example of reviewer's comments

This is an example of our response.

> This is an example of quoted texts from the updated manuscript

Reviewer #1: The authors have done a thorough job responding to my comments, and I believe the whole paper is much improved.

Thank you very much.

Just one new substantive issue has arisen during this revision: the results reported for the SBayesR method are very poor, and seem to strongly contradict the original publication on this method. Indeed, it is a bit hard to believe that it performs quite so poorly, and the reasons for its poor performance need to be understood and either corrected or explained. For example, for height, SBayesR does no better than just Age + Sex in predicting height - so it essentially has a 0% R2 when you consider the genetic component only. In contrast, LLoyd-Jones et al report that SBayesR achieved an R2 of >35% for height in the UK biobank. Something is clearly wrong, either with the SBayes software or with the way it has been applied. (Other traits show a similar pattern, but the height result is particularly striking.)

Of course, I don't know what the problem is, but I suggest a first step would be to ask the SBayesR authors if they have suggestions, and/or get their original code and see if you can reproduce their published results.

Thank you very much for raising the potential issue with SBayesR. We followed the suggestion and contacted the SBayesR authors. With their help, we were able to locate the source of the issue and have updated the results in the revised manuscript.

Specifically, our analysis confirmed the following:

1. In our SBayesR analysis presented in the previous revision, we computed the genome-wide sparse LD matrix by splitting the genome into small "chunks" of at most

5,000 SNPs, following the SBayesR tutorial on the GCTB website (https://cnsgenomics.com/software/gctb/#Tutorial). We subsequently merged those 143 chunks into one genome-wide LD matrix file.

2. With help from the authors of SBayesR, we were able to identify that this “merge” operation does not fully-support the inter-chromosomal merge. In fact, the LD matrix used in our initial SBayesR analysis was corrupt and that explains why we saw low predictive performance of SBayesR in the previous revision.
3. We re-generated the genome-wide LD matrix by performing the two-pass merge to mitigate the issue: one-pass for the intra-chromosomal merge to generate chromosome-wide LD matrices for all autosomes and the other pass to combined those LD matrices into one genome-wide LD matrix.
4. Using the new LD matrix, we fit the Bayesian regression models. We changed one of the MCMC parameters, the number of burn-in iterations, from 2000 to 4000 so that we have the exact same configurations as in the original SBayesR publication. We also included the variants in the MHC region following your advice (described below in response for “other items”)

After solving the issue, we see an improved predictive performance from SBayesR.

We would like to emphasize that the authors of SBayesR/GCTB were very helpful and provided invaluable feedback to identify the source of the issue. For example, they have expanded the FAQ section in their SBayesR/GCTB website (https://cnsgenomics.com/software/gctb/#FAQ) and performed additional computation and provided LD matrix files that helped us to identify the source of the issue. We have updated the acknowledgement section in our manuscript.

While we were updating the SBayesR analysis, we realized another potential issue in the original performance comparison presented in the earlier version of the manuscript. Specifically, some methods, such as SBayesR and PRS-CS, were trained on the 80% of the unrelated white British UKB sample (combined sets of training and validation set) whereas other methods, such as snpnet and its elastic net extension, were trained on the 60% of the unrelated white British UKB sample (the training set) to optimize hyper parameters such as the lambda value in lasso regression. To address this issue and provide benchmarking results from a fair comparison, we performed additional experiments to evaluate the lasso/elastic-net performance with refitting. In particular, after choosing the optimal tuning parameter(s) based on the validation set, we refit the lasso/elastic-net on the combined training/validation set under that optimal parameter(s) and evaluate the test performance with the refitted model. By applying this “refit” procedure, we were able to compare the predictive performance of different models, all of which are trained on the same 80% of the unrelated white British UKB sample (the combined training and validation set). The comparison results are detailed in Table 3, 7, 9, 11 respectively for the four phenotypes.

Here is an example table for height:

**Model**	**Test set R^2**	**Model Size**
Lasso	0.7134	45,653
Elastic Net	0.7128	45,549
Ridge	0.6986	175,012
PRS-CS	0.5615	148,064
SBayesR	0.7019	667,057
P + T	0.5912	15,544
Clumping	0.6181	17,433

Other items:

in SBayes i noticed you excluded the MHC. Maybe this is recommended by SBayes software, but it seems likely to hurt R2 and AUC for many traits as the MHC has a strong effect on many traits. To make results comparable across methods it seems necessary to either exclude or include MHC for all methods. (It seems unlikely that this issue explains the poor performance on height noted above.)

Thank you very much for your suggestion. To see whether the inclusion of the MHC region would help improve the predictive performance, we have performed the SBayesR analysis with and without the MHC region. As you can see below, the inclusion of the MHC region improved the predictive performance. In the revised manuscript, we presented SBayesR results with the MHC region. Note that all the results presented in the updated manuscript include the MHC region.

Model	Test set R^2		Test set AUC	
	Standing height	BMI	Asthma	High cholesterol
SBayesR with MHC	0.7019	0.1251	0.6320	0.7327
SBayesR without MHC	0.7012	0.1242	0.6278	0.7324

Reviewer #2: my previous comments were minor and the authors have addressed these comments.

Thank you very much for your time and feedback on our manuscript.
